# Ce Promotion of In_2_O_3_ for Electrochemical Reduction of CO_2_ to Formate

**DOI:** 10.1021/acscatal.4c02619

**Published:** 2024-10-25

**Authors:** Tim Wissink, Floriane A. Rollier, Valery Muravev, Jason M.J.J. Heinrichs, Rim C.J. van de Poll, Jiadong Zhu, Dimitra Anastasiadou, Nikolay Kosinov, Marta C. Figueiredo, Emiel J.M. Hensen

**Affiliations:** † Laboratory of Inorganic Materials and Catalysis, Department of Chemical Engineering and Chemistry, Eindhoven University of Technology, P.O. Box 513, 5600 MB Eindhoven, The Netherlands

**Keywords:** CO_2_ electroreduction, formate, In_2_O_3_, dopants, cerium promotion

## Abstract

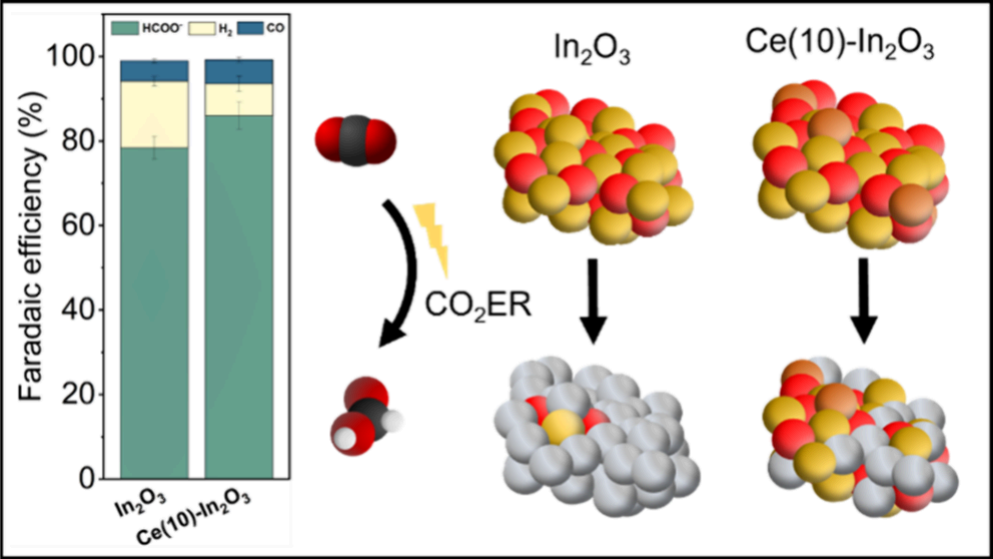

In_2_O_3_ is a promising electrocatalyst for CO_2_ electroreduction (CO_2_ER) to formate. In_2_O_3_ nanoparticles doped with Pd, Ni, Co, Zr, and Ce promoters using flame-spray pyrolysis were characterized and evaluated in a gas diffusion electrode for the CO_2_ER. Doping results in slight shifts of the In binding energy as probed by XPS, which correlates with a change of the Faradaic efficiency to formate (FE_formate_) in the order Ce-doped In_2_O_3_ > Zr-doped In_2_O_3_ > In_2_O_3_ > Pd-doped In_2_O_3_ > Ni-doped In_2_O_3_ > Co-doped In_2_O_3_. However, the differences in CO_2_ER performance are caused mainly by the different extent of In_2_O_3_ reduction. Co-doped In_2_O_3_ is prone to complete reduction to a stable Co–In alloy with a low FE_formate_ due to a high hydrogen evolution activity. The stabilizing effect of Ce on In_2_O_3_ is further demonstrated by an X-ray absorption spectroscopy study of a set of Ce-doped In_2_O_3_ samples (10, 50, 90 at%), highlighting that reduction of In_2_O_3_ is suppressed with increasing Ce content. Optimum performance in terms of FE_formate_ is obtained at a Ce content of 10 at%, which is attributed to the stabilization of In_2_O_3_ under negative bias up to −2 V. At higher Ce content, less active CeO_2_ is formed. The highest FE_formate_ of 86% observed for In_2_O_3_ doped with 10 at% Ce, at a current density of 150 mA/cm^2^, compares favorably with a FE_formate_ of 78% for In_2_O_3_.

## Introduction

CO_2_ is considered a circular carbon source for the chemical industry and transport sector. Converting CO_2_ to chemicals and fuels using renewable energy sources could replace fossil resources in these sectors, thereby closing the carbon cycle. Electrochemical CO_2_ reduction offers a direct pathway to utilize renewable electricity to convert CO_2_ into base chemicals, which can be further converted to other chemicals and fuels.^1^ Typical products of the CO_2_ electroreduction reaction are CO, ethylene, ethanol, and formic acid.^2^ Among these, formic acid and formate have the potential to serve as feedstock for chemicals and as a liquid hydrogen carrier relevant to energy storage in chemical bonds.^3,4^ Electrochemical CO_2_ reduction to formate can achieve high Faradaic efficiency at industrially relevant current densities, making it one of the most promising electrochemical processes for commercialization.^1,5^ Typical electrocatalysts for CO_2_ reduction comprise the metals Sn, In, and Bi.^2,5,6^ Such catalysts reach Faradaic efficiencies close to 100% at current densities >100 mA/cm^2^.^5^

The reaction mechanism of CO_2_ electroreduction (CO_2_ER) to formic acid involves an oxygen-bound formate intermediate on the catalytic surface.^7^ The carbonyl pathway that leads to undesired CO byproduct competes with this formate pathway. A high oxophilicity of a metal catalyst, therefore, stabilizes the formate intermediate, which is thought to increase the selectivity of the formate pathway over the carbonyl one.^8^ On the other hand, metal oxides are widely believed to be the most active for CO_2_ER to formate. Still, their stability against reduction to the metal under the reducing CO_2_ER conditions is heavily debated.^9−13^ Several theoretical studies showed that defects in metal oxides, such as oxygen vacancies, can act as CO_2_ adsorption sites.^14,15^ Alternatively, it is proposed that surface hydroxyls can react with CO_2_ to form a carbonate intermediate before^15,16^ or instead of the formate intermediate.^17^ A major benefit of metal oxides over metallic catalysts is the higher kinetic barrier of the former for the main competing hydrogen evolution reaction (HER).^18,19^ Experimental studies to understand the role of oxide surfaces indicate that oxygen vacancies and hydroxyls are involved in reducing CO_2_ to formate. Nevertheless, these studies are primarily conducted at low current densities in H-cell configurations employed in conventional electrochemical studies, which are, for instance, more conducive to coupling with surface-sensitive characterization techniques such as infrared spectroscopy.^16,17,20−22^ Industrial applications require high current densities, implying operation at more negative potentials. Convincing experimental evidence for the importance of oxides as the main catalyst during the CO_2_ER at industrially relevant conditions is lacking. Broekmann and co-workers utilized operando Raman, X-ray absorption spectroscopy (XAS), and X-ray diffraction (XRD) to study the impact of experimental conditions at which Sn- and Bi-oxide phases remain stable utilizing a gas diffusion electrode (GDE) configuration.^11,13,23^ They observed a strong correlation between the presence of Sn-oxide and Bi-oxy-carbonate phases and high Faradaic efficiency (FE) toward formate. Such a study has not been undertaken yet for In-based CO_2_ER catalysts. Wang et al. showed by operando XAS that a mixed In–Sn-oxide shell was maintained on an In–Sn alloy electrocatalyst during CO_2_ER at a potential up to −1.2 V vs RHE in an H-cell configuration.^24^

Here, we report on In_2_O_3_-based catalysts for CO_2_ electroreduction to formate. In_2_O_3_ nanoparticles doped with Pd, Ni, Co, Ce, and Zr were obtained using flame synthesis. It has been demonstrated that flame spray pyrolysis (FSP) is suitable to incorporate other elements in In_2_O_3_ nanoparticles.^25^ Doping of the In_2_O_3_ with these transition metals can modify the catalytic properties of the In_2_O_3_ surface and its stability against metal reduction, thereby impacting the CO_2_ER to formate in terms of FE and stability. Pd and Co were chosen because of their activity toward reduction of CO_2_ to formate at low potential.^26,27^ Ce and Zr were selected, as their oxides form strong interactions with In_2_O_3_,^28^ which can stabilize their dispersion and impact oxygen vacancy formation as reported to be beneficial for thermal CO_2_ hydrogenation.^25,29^ During our investigations, we found that Zr and especially Ce doping positively impacted the FE to formate. This led us to investigate in more detail mixed oxides of Ce and In prepared by FSP by varying the Ce content from 0% to 90%. The catalytic performance of the metal oxides is evaluated using gas diffusion electrodes at industrially relevant current densities in the range of 100–200 mA/cm^2^. The samples are investigated by electron microscopy, XRD, XAS, and X-ray photoelectron spectroscopy (XPS) to elucidate the promoters’ influence on the catalytic activity and stability of In_2_O_3_ as a catalyst for the CO_2_ER. This work is also available as part of a PhD thesis.^30^

## Methods

### Catalyst Preparation

#### In_2_O_3_ Nanoparticles

Nanoparticles (NPs) of In_2_O_3_ were prepared by flame spray pyrolysis (FSP) using a Tethis NPS10 setup. A 0.15 M In(NO)_3_ solution was prepared by dissolving an appropriate amount In(NO)_3_·5H_2_O (99%, Alfa Aesar) in an equivolumetric mixture of ethanol (99.9%, Biosolve) and 2-ethylhexanoic acid (99%, Sigma-Aldrich). This synthesis was modified by introducing dopants during the FSP preparation, namely 5 wt % Co(NO_3_)_2_·6H_2_O (98%, Sigma-Aldrich), 5 wt % Ni(NO_3_)_2_·6H_2_O (98.5%, Sigma-Aldrich), 5 wt % Pd(OCOCH_3_)_2_ (98%, Sigma-Aldrich), 5 wt % Zr(acac)_2_ (99.9%, Sigma-Aldrich), and 10–90 at% Ce(acac)_2_ (acac = acetylacetonate, 99.9%, Sigma-Aldrich). For comparison with XPS analysis, the weight-based concentration of the dopant is converted to the atomic concentration with respect to total metal content as listed in Table 1 and Table 2. The solution was then injected into the nozzle of the FSP setup using an injection rate of 5 mL/min. The flame was fed with a flow of 3.0 L/min O_2_ and 1.5 L/min CH_4_, to which a dispersion flow of 5.0 L/min O_2_ was added. The solid particles were collected from a quartz filter placed downstream of the flame region. The (doped) In_2_O_3_ solids were sieved over a 250 μm steel sieve. The Ce–In-oxide catalysts are denoted by their atomic Ce content, i.e., Ce(50)-In_2_O_3_ having a Ce content of roughly 50 at% with respect to total of In and Ce content.

**Table 1:** Physicochemical Properties of (Doped) In_2_O_3_ Nanoparticles Synthesized by FSP

	Loading dopant			Particle size		XPS analysis	
Catalyst	Nominal (wt%)^a^	Nominal (at%)^a^	Surface (at%)^b^	d_TEM_ (nm)	d_XRD_ (nm)	In_2_O_3_ (at%)	In(OH)_3_/defects (at%)
In_2_O_3_	–	–	–	6.2 ± 1.4	9.2	87	13
Zr–In_2_O_3_	4.9	7.5	5.7	6.3 ± 1.5	8.3	85	15
Ce–In_2_O_3_	9.6	9.7	11	6.0 ± 1.6	8.2	94	6
Pd–In_2_O_3_	5.3	6.8	7.7	5.8 ± 1.7	9.0	92	8
Ni–In_2_O_3_	5.0	11.0	16	7.3 ± 1.5	9.1	82	18
Co–In_2_O_3_	5.0	11.1	11	6.7 ± 1.6	9.3	64	36

a ICP elemental analysis.

b XPS surface analysis.

**Table 2:** Physicochemical Properties of Mixed Ce–In-Oxide Nanoparticles Synthesized by FSP

	Ce loading		Particle size			XPS analysis		
Catalyst	Nominal (at%)^1^	Surface (at%)^2^	d_TEM_ (nm)	d_XRD_ In_2_O_3_ (nm)	d_XRD_ CeO_2_ (nm)	In_2_O_3_ (at%)	In(OH)_3_/defect (at%)	Ce^4+^/Ce^3+^ ratio
In_2_O_3_	–	–	6.8 ± 2.0	8.6	–	80	20	–
Ce(10)-In_2_O_3_	11.0	8.0	6.2 ± 1.5	7.8	–	92	8	3.2
Ce(50)-In_2_O_3_	51.5	38	6.6 ± 1.7	–	4.6	89	11	4.2
Ce(90)-In_2_O_3_	92.6	83	6.0 ± 2.0	–	7.1	55	45	5.3

#### Gas Diffusion Electrode (GDE) Preparation

A suspension of the catalyst was prepared by weighing appropriate amounts of (doped) In_2_O_3_, Vulcan XC-72R activated carbon (AC) as support and PTFE nanoparticles (Sigma-Aldrich, 1 μm) and dispersing these solids by sonication in 3 mL isopropanol (99.9%, Sigma-Aldrich) and 1 mL ultrapure water. Nafion ionomer solution (D-520, dispersion, 5% w/w in water and isopropanol, Alfa Aesar) was added to reach a mixture containing 20 wt % Nafion with respect to solid particles. The solution was sonicated for ∼30 min. The ink was sprayed on a commercial gas diffusion layer (GDL, ELAT LT1400W, FuelCellStore) with a spray gun. The total loading of the catalyst on the GDE, as determined by weighing the GDL before and after catalyst deposition, amounted to 0.30 ± 0.06 mg/cm^2^ for the (doped) In_2_O_3_ nanoparticles and 0.47 ± 0.07 mg/cm^2^ for the mixed Ce–In catalysts. For operando XAS analysis, a GDE was prepared without activated carbon and PTFE particles employing a higher catalyst loading of 1.04 ± 0.12 mg/cm^2^ on a commercial GDL (Sigracet 22BB, Ion-Power).

### Characterization

#### Inductively Coupled Plasma Optical Emission Spectrometry (ICP-OES)

The elemental composition of the FSP-synthesized nanoparticles was determined using an AMETEK ICP optical emission spectrometer (Spectroblue) with axial plasma viewing, equipped with a free-running 27.12 MHz generater (1400 W). Around 25 mg of the sample was dissolved in 5 mL concentrated H_2_SO_4_ at 220 °C. After cooling, the solution was diluted to 50 mL with demineralized water. Two solutions were prepared to perform the measurements in duplicate. A calibration line with concentrations between 0 to 20 mg/L In, 0 to 10 mg/L Ce, 0 to 5 mg/L Zr, and 0 to 3 mg/L for Co and Ni was used. A second calibration line with concentrations between 0 and 5 mg/L Pd was used due to the overlap of the emission lines of Pd and In.

#### X-ray Photoelectron Spectroscopy (XPS)

XPS was performed with a K-Alpha XPS apparatus (Thermo Scientific) using an Al anode (Al Kα monochromatic irradiation, 1486.6 eV) operating at 72 W. A spot size of 400 μm was used. Survey and core level spectra were recorded at pass energies of 200 and 50 eV, respectively. The background pressure inside the analysis chamber was kept below 8 × 10^–8^ mbar. During measurements, a maximum pressure of 3 × 10^–7^ mbar was observed due to using low energy Ar^+^ ions for charge neutralization. Catalyst powders were pelletized before XPS analysis to prevent the charging of the insulating In_2_O_3_ particles. All spectra were energy corrected by the U‴ component at 916.7 eV of Ce.^31^ After energy correction of the Ce-containing catalysts, the resulting C 1s peak position of adventitious carbon was used to correct the energy scale for the samples that did not contain Ce. The core level spectra were deconvoluted using a Shirley background and using GL(30) line shapes for most contributions. The metallic In peak was fitted using an LA(1.1,1.7,3) line shape obtained from an In reference sample, which was etched in the vacuum chamber of the XPS apparatus (Figure S8a). The Ce 3d core-line spectra were fitted according to the literature.^31−33^

#### X-ray Absorption Spectroscopy (XAS)

Operando XAS analysis was performed at the ROCK beamline at the SOLEIL synchrotron (Paris, France). Data was collected in fluorescence mode at the In K-edge (27940 eV). An electrochemical cell developed in-house by SOLEIL was used. The GDE prepared as described above was used as the working electrode. A gas flow chamber with a Kapton window was placed at the back of the working electrode, while the catholyte chamber was in front of the working electrode. The cell was positioned at a 39° angle with respect to the incoming X-ray beam, and the fluorescence signal was measured from the back of the electrode by an avalanche-photodiode fluorescence detector positioned perpendicular to the beam. A Nafion proton exchange membrane (N324, Ion Power) was used to separate the catholyte and anolyte chambers. Pt foil and Ag/AgCl (6 mm, redoxme, measured at 0.23 V vs SHE at pH 1) electrodes were used as counter and reference electrodes, respectively. An IviumStat potentiostat was used to set the potential and record the electrochemical response of the working electrode. The anolyte (0.5 M H_2_SO_4_) was pumped using a peristaltic pump at a flow of 12 mL/min. Catholyte (0.5 M KHCO_3_) was introduced in the catholyte chamber. A mass spectrometer (MKS Cirrus LM99 Analyzer) was used to detect CO_2_, H_2_ and CO in the gas exit line from the gas chamber at the back of the GDE. Catholyte was not pumped around to avoid leakage through the GDE into the gas exit toward the mass spectrometer. The catholyte was refreshed in each experiment. CO_2_ was passed at the back of the GDE at a flow rate of 5 mL/min (flow-by mode). A spectrum was recorded every 0.5 s. As 10 spectra were averaged to increase the signal-to-noise ratio, the temporal resolution of the measurements was 5 s. Energy calibration was done using an In foil. The X-ray absorption near-edge structure (XANES) data were background-subtracted, normalized, and fitted using linear combination analysis with XAS viewer as implemented in the Larch software package. The extended X-ray adsorption fine structure (EXAFS) analysis on k^3^-weighted In K-edge data was also performed with XAS viewer. Scattering paths were calculated with FEFF8 using crystal structures of In_2_O_3_, In and In(OH)_3_. The amplitude reduction factor S_0_^2^ (0.77) used for fitting the Ce–In-oxide EXAFS spectra was determined by fitting the two In–In scattering paths of the first shell of reference In foil, fixing the coordination numbers to, respectively, 4 and 8 (Table S2).

#### X-ray Diffraction (XRD)

XRD was performed using a Bruker D2 Phaser diffractometer using Cu Kα radiation (1.5406 Å). The XRD diffractograms of catalyst powders were recorded between 2θ = 10° and 90° at a scan rate of 1 s/step and a step size of 0.02°. XRD patterns on a GDE were obtained at a scan rate of 2 s/step and a step size of 0.01°. The crystallite size was calculated using the Scherrer equation as implemented in the DIFFRAC.EVA software package.

#### Wide Angle X-ray Scattering (WAXS)

Ex situ WAXS measurements were performed at beamline ID31 of the ESRF synchrotron (Grenoble, France). The incident photon energy was 75 keV (l = 0.0165 nm) using a Pilatus CdTe 2 M detector set up in the Debye–Scherrer geometry. Kapton tubes were filled with powder samples and sealed with bee wax before analysis. The WAXS peaks were fitted with a Voigt function to determine the full width at half-maximum (fwhm). The average crystal size was calculated using the Scherrer equation. Rietveld refinement was performed using GSAS-II software. In_2_O_3_ and CeO_2_ crystal structures were used to fit the diffraction patterns of the as-prepared catalysts. The patterns were fitted between 2θ = 1.5 and 9. The background was fitted using a chebyschev-1 function with 3 coefficients. Instrument parameters were determined by fitting a reference CeO_2_ sample and fixed for all samples. Sample displacement parameters were determined by fitting the pure In_2_O_3_ sample and fixed for all Ce-doped In_2_O_3_ samples. The fitting was performed by subsequent fitting of the phase fractions, micro strain and lattice parameter in the given order. After several iterations (>3), the size and lattice parameters were fitted simultaneously. The fit of the Ce(50)-In_2_O_3_ sample was optimized by subsequent fitting of the atom fractions, atom displacement and thermal displacement.

#### Transmission Electron Microscopy (TEM)

Transmission electron images were obtained using an FEI Tecnai (type Sphera) instrument operating at an acceleration voltage of 200 kV and an FEI cryoTITAN instrument operating at an acceleration voltage of 300 kV. Catalyst particles were dispersed in ethanol via ultrasonication and deposited on a holey Cu support grid. The particle size and lattice constants of the catalysts were obtained using the software ImageJ.

#### Scanning Transmission Electron Microscopy Energy-Dispersive X-ray Spectroscopy (STEM-EDX)

The distribution of In and Ce in the In_2_O_3_–Ce samples was studied using STEM-EDX. Measurements were carried out on a FEI cubed Cs-corrected Titan operating at 300 kV. In_2_O_3_–Ce samples were dispersed in ethanol via ultrasonication and deposited on a Cu support grid with a holey carbon film. Elemental analysis was done with an Oxford Instruments EDX detector X-Max^N^ 100TLE.

### Electrochemical Experiments

#### Electrocatalytic CO_2_ Reduction

In flow-through mode, CO_2_ electroreduction was performed in a flow cell with an electrode with a geometric surface area of 1 cm2 (Micro Flow Cell, ElectroCell). A Nafion proton exchange membrane (N324, Ion Power) separated the catholyte and anolyte chambers. The Nafion membrane was pretreated in a 10 vol % H_2_O_2_ solution (diluted from 33 vol % solution, VWR Chemicals) at 80 °C for 1 h and subsequently in a 3 M H_2_SO_4_ (≥95%, Merck) solution (80 °C, 1 h). An Autolab 302N potentiostat was used for electrochemical analysis for cyclic voltammetry and chrono-potentiometry measurements. A three-electrode system was used with a Pt mesh as a counter electrode and a leakless Ag/AgCl electrode (1.6 mm, EDAQ, measured at 0.37 V vs SHE at pH 1) as the reference. The as-prepared GDEs were used as working electrodes. Potentials are specified against the Ag/AgCl reference electrode. As catholyte, 0.5 M KHCO_3_ solution was prepared by dissolving high purity KHCO_3_ (analysis grade, Merck) in ultrapure water (18.2 MΩ·cm). As anolyte, 0.5 M H_2_SO_4_ solution was used by diluting H_2_SO_4_ (>95%, Sigma-Aldrich) in ultrapure water. The catholyte solution was degassed by N_2_ and saturated with CO_2_ before use. The backside of the GDE was supplied with a gaseous flow of 10 mL/min CO_2_. The catholyte and anolyte liquid flows were set at 50 mL/min. Cyclic voltammetry and galvanostatic measurements were performed for 1 h at a current density of 200 mA/cm^2^ during and after which the catholyte was analyzed for products. Faradaic efficiencies toward formate were calculated from the ratio of charge that is needed to produce the obtained formate over the total amount of charge that was consumed at the cathode:
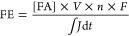
with [FA] being the concentration of formate in the cathode compartment, *V* the catholyte volume, *n* the number of consumed electrons (two) per formate molecule, *F* Faraday’s constant, *J* the current density, and *t* time. The experiments were repeated at least twice with a fresh GDE with the same loading to estimate the experimental error. The current densities are reported based on the exposed geometrical surface area of the GDE. This study uses the Faradaic efficiency and current density as major indicators for the catalyst performance and is used to analyze and optimize the cathodic catalyst. As pointed out by, e.g., the group of Seger and co-workers,^34,35^ for industrial applications, the system also needs to be optimized for CO_2_ conversion efficiency, which is considered outside the scope of this work.

#### High-Performance Liquid Chromatography (HPLC)

Dissolved reduction products were analyzed by HPLC. A Shimadzu HPLC containing a Polar C18 column (Luna Omega 3 μm, 150 × 3.0 mm) was used to determine the formic acid concentration. A potassium phosphate buffer (pH 2.0) was used as eluent, which was prepared by dissolving 1.4150 g KH_2_PO_4_ (≥99%, Sigma-Aldrich) and 1.6854 g H_3_PO_4_ solution (85%, Merck) in 1.0 L ultrapure water. The column oven temperature was set at 30 °C and the UV–vis detector cell temperature at 40 °C. Samples were taken from the catholyte by diluting 100 μL of electrolyte to 1.0 mL with 900 μL 0.1 M H_3_PO_4_ solution (1:10) to convert bicarbonate to CO_2_. An injection volume of 10 μL and a total eluent flow of 500 μL/min was used. The retention time of the formic acid peak was about 2 min. Reference solutions from 263.434 mM down to 5.268 mM formic acid (≥98%, Sigma-Aldrich) were used to obtain a calibration curve for HPLC analysis. Two stock formic acid solutions (263.434 mM and 105.60 mM) were prepared by diluting 1.0 mL formic acid to 100 and 250 mL in 0.5 M KHCO_3_ in ultrapure water and were further diluted for reference solutions. The calibration solutions were also prepared by 1:10 dilution in 0.1 M H_3_PO_4_ (85%, Merck) and analyzed in triplicate. Peak integration was performed using the ICIS algorithm included in the Shimadzu software package.

#### ^1^H Nuclear Magnetic Resonance Spectroscopy (^1^H NMR)

Dissolved reaction products were analyzed by ^1^H NMR spectroscopy. NMR spectra were recorded using a 400 MHz Bruker spectrometer. An aliquot of 450 μL was taken directly from the catholyte, followed by the addition of 50 μL of D_2_O (99.9%, Sigma-Aldrich) containing 10 mM DMSO (>99.9%, Biosolve) and 50 mM phenol (≥99%, Sigma-Aldrich) as internal standards. Standard solutions for HPLC calibration were used to verify the DMSO and phenol concentrations. The NMR signal due to protons of water was suppressed by a solvent presaturation sequence. The signal-to-noise ratio was improved by averaging 48 scans with a 16 s delay time. The resulting spectra were calibrated with reference to the DMSO peak position, set at 2.600 ppm in accordance with Kuhl et al.^36^ Then, the peak belonging to the carbon bonded proton in formic acid was observed at 8.33 ppm.

#### Gas Chromatography

Gaseous products were analyzed online using a TRACE 1300 gas chromatograph (Thermo Fisher). The permanent gases (H_2_, CO, CO_2_) and the hydrocarbons (methane, ethylene) were analyzed on separate channels. A Hayesep Q precolumn and a Shin-Carbon ST column with TCD were used for permanent gas analysis. An Al_2_O_3_/KCl column with FID was used to separate the light hydrocarbons.

## Results and Discussion

### Catalyst Characterization

In_2_O_3_ nanoparticles and In_2_O_3_ nanoparticles doped with Zr, Ce, Pd, Ni, and Co were obtained by FSP. Their physicochemical properties are summarized in Table 1.

The Zr, Pd, Ni and Co contents were targeted at 5 wt % with respect to the total weight of the particles, while the content of the Ce dopant was aimed at 10 wt %. The atomic and weight loadings of the promoters are given in Table 1. The average size of the In_2_O_3_ nanoparticles determined by TEM was 6.2 ± 1.4 nm (Figure S1). The size of the doped In_2_O_3_ nanoparticles was close to that of pure In_2_O_3_ with the average particle size falling in the range of 5.8–7.3 nm with a nearly dopant-independent size distribution. This shows that doping did not strongly affect the particle size (Figure S1). Both In_2_O_3_ and doped In_2_O_3_ particles are made up of cubic In_2_O_3_ as follows from the XRD patterns in Figure S2. The dopants are highly dispersed, as seen from the absence of other diffraction lines. This suggests that the promoters are doped into the In_2_O_3_ lattice, albeit they can also be present as very small oxide particles.^37^ The shift of the In_2_O_3_ diffraction lines for the Ce–In_2_O_3_ sample toward lower angles indicates lattice expansion by incorporating Ce ions in the In_2_O_3_ crystal structure. The radii of Ce^4+^ and Ce^3+^ ions of respectively 101 and 115 pm are larger than the radius of 81 pm of In^3+^. The cations of the other dopants in their most likely oxidation states are too similar to that of In^3+^ to judge their incorporation in the In_2_O_3_ lattice from XRD.^25,38^ The average crystal sizes of the (doped) In_2_O_3_ nanoparticles calculated by the Scherrer equation are also listed in Table 1. The crystallite sizes of the In_2_O_3_ particles doped with Pd, Ni and Co are very similar to that of In_2_O_3_ (∼9 nm), while those for the Zr- and Ce-doped In_2_O_3_ particles are slightly smaller (∼8 nm).

**Figure 1:**
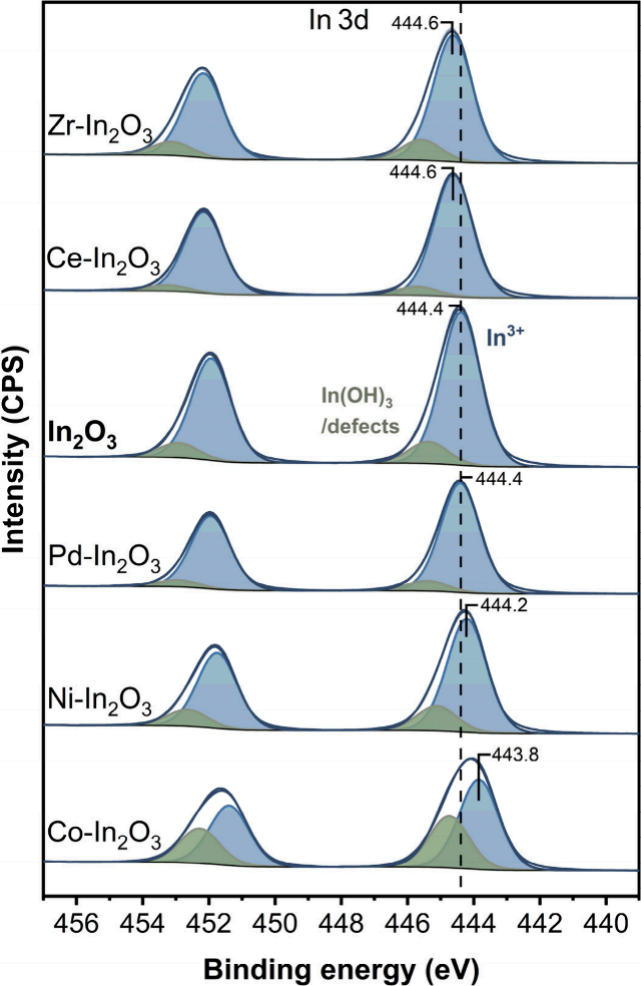
XPS spectra of the In core-line region of the FSP-synthesized doped In_2_O_3_ catalysts. All spectra were energy corrected by the U‴ component at 916.7 eV of cerium. The resulting C 1s binding energy (measured at 285.1 eV) was used to energy correct spectra of samples that contain no cerium.

XPS was used to analyze the surface composition of the samples. The surface concentration of the dopants is determined from the core level XPS spectra of In and the dopants. The results are listed in Table 1. The surfaces of Pd- and Ni-doped In_2_O_3_ are slightly enriched in dopant compared to their nominal contents. While the Zr-doped In_2_O_3_ nanoparticles contain slightly less Zr at the surface than the nominal content, the bulk and surface dopant contents for Ce- and Co-doped In_2_O_3_ are nearly the same. Overall, the minor differences observed point to a high dispersion of the dopant with some indications of dopant enrichment in the surface for Ni and Pd. For a similarly prepared Ni-doped In_2_O_3_ catalyst used in CO_2_ hydrogenation, it was found that Ni is highly dispersed as Ni cations in the bulk and at the surface of In_2_O_3_.^39^ The group of Pérez-Ramirez also used FSP to prepare a range of metal-doped In_2_O_3_ nanoparticles and found that most metal dopants were evenly distributed in the bulk and surface of In_2_O_3_.^25^Figure 1 shows the In 3d XPS spectra of the In_2_O_3_ and doped In_2_O_3_ samples. The spectra consist of two main features representing the In 3d_5/2_ and In 3d_3/2_ states. Two contributions are included to fit these In spectra. The main peak at an In 3d_5/2_ binding energy of 444.4 eV is attributed to In_2_O_3_,^40^ The shoulder, with a higher binding energy of 445.4 eV, is due to In-hydroxide or defects in In_2_O_3_ (Figure S3).^40^ Doping with Ce and Zr results in a small shift (0.2 eV) of the binding energy of the In 3d_5/2_ peaks. The main In 3d_5/2_ binding energy shifts from 444.4 to 444.6 eV, as was also observed for Zr doping by Pinheiro Araújo and co-workers.^41,42^ This shift indicates a higher effective charge on the In ions, which can be caused by transfer of electrons from In to Ce and Zr.^43^ The contribution of In(OH)_3_/In_2_O_3_-defects does not change upon Zr doping and decreases for Ce. An opposite shift in the binding energy is observed when In_2_O_3_ is doped with Ni and Co, resulting in a shift of the main In 3d_5/2_ peak to respectively 444.2 and 443.8 eV. The Co-doped In_2_O_3_ sample contains a higher contribution of In(OH)_3_/In_2_O_3_-defects of 36% as compared to the contribution of 13% for In_2_O_3_. This, together with the relatively large binding energy shift of In and the homogeneous distribution of Co in the In_2_O_3_ particles, indicates the well-mixed nature of the Co–In-oxides as observed previously for FSP-prepared Co-doped In_2_O_3_.^25^ There are no significant changes in the In 3d spectra upon introducing Pd in In_2_O_3_. The same trends in binding energies are observed when comparing the O 1s spectra (Figure S4). The O 1s binding energy of lattice oxygen in In_2_O_3_ is observed at 529.9 eV and shifts to 530.1 eV upon doping with Ce and Zr, while a slight shift to lower energies of 529.7 and 529.5 eV is observed for the Ni- and Co-doped In_2_O_3_, respectively.

The XPS spectra of the dopants were deconvoluted, as shown in Figure S5. The Ce content in the In_2_O_3_ particles is 9.7 at%, close to the nominal Ce content (Table 1). Deconvolution of the Ce 3d spectrum of the Ce-doped In_2_O_3_ using contributions of Ce^3+^ and Ce^4+^ reveals a substantial amount of 24% Ce^3+^ (Figure S5a).^37,44^ The existence of Ce in the +3 oxidation state suggests the presence of oxygen defects.^45^ Ce^3+^ might be included in the lattice of In_2_O_3_ as supported by the shift of the XRD diffraction lines in Figure S2.^46,47^ Ce^4+^ could also substitute In^3+^ in the lattice of In_2_O_3_,^46^ but the presence of a significant amount of Ce^4+^ can most likely be attributed to the presence of small CeO_2_ particles, which can also generate Ce^3+^.^45^ The Co 2p_3/2_ region was fitted using a model from the literature (Figure S5d).^48,49^ The satellite feature at 786 eV indicates the predominant presence of CoO and Co(OH)_2_ and, in line with this, the Co 2p_3/2_ region could be fitted by contributions due to Co^2+^ at 782 and 780 eV. A minor feature at a lower binding energy of 779.3 eV can be attributed to fully oxidized Co_3_O_4_.^48^ These results are in line with previous reports where Co forms small CoO_*x*_ clusters and dopants in FSP-synthesized In_2_O_3_.^25^ The Ni 2p_3/2_ core-line region spectrum contains a main peak at 855.5 eV and a broad satellite feature at 861 eV (Figure S5c). A small feature at 854 eV indicates the minor presence of NiO.^39^ The main peak at 855.5 eV has been attributed to Ni^3+^ before,^50^ but this assignment is not unequivocal. Based on reference spectra, it could also be assigned to Ni(OH)_2_,^50,51^ but a more likely explanation is that it represents small NiO clusters in strong interaction with In_2_O_3_.^39^ The Pd 3d core-line region spectrum reveals a large variety of Pd species. Besides the presence of PdO and a feature assigned to oxygen-deficient PdO, some metallic Pd is also present (Figure S5e).^52−54^ Overall, XPS indicates that the dopants cause minor (Ce–In_2_O_3_) to significant (Co–In_2_O_3_) shifts in the In 3d binding energy, indicative of their introduction in the In_2_O_3_ lattice. Nevertheless, it is also clear that part of the dopants are present as separate oxide phases. Regarding doping, Zr and Ce cause a slight increase of the In 3d binding energy for In_2_O_3_ of 0.2 eV, reflecting a slightly higher positive charge of In^3+^.^41^ Doping with Ni and Co results in an opposite shift toward lower binding energies.

### CO_2_ Electroreduction

**Figure 2:**
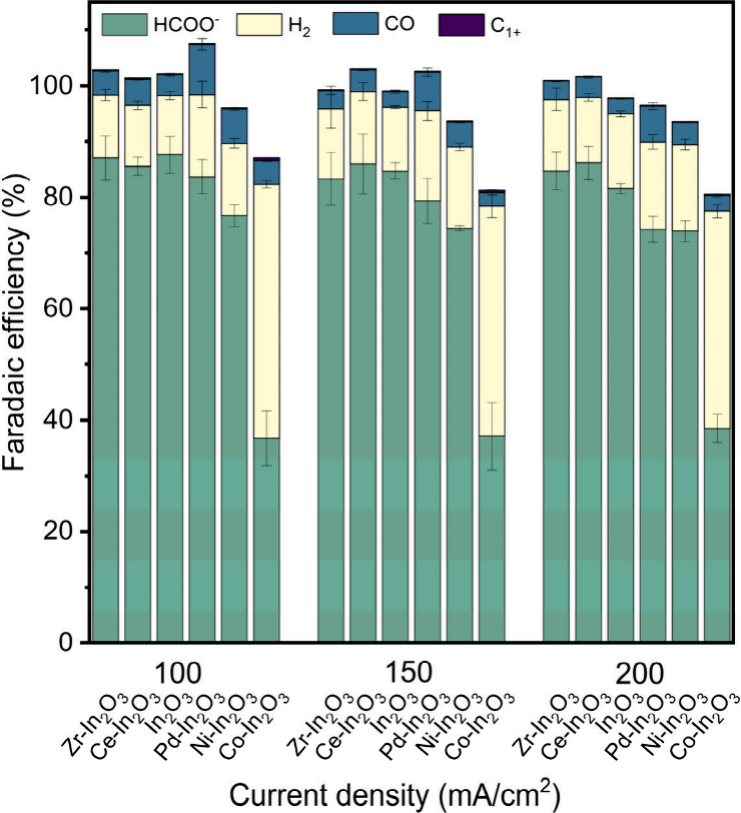
Faradaic efficiency as a function of current density on doped In_2_O_3_ catalysts after 1 h CO_2_ER in 0.5 M KHCO_3_. Catalysts nanoparticles are deposited on a GDE (0.30 ± 0.06 mg/cm^2^). Experiments were done in triplicate (Note: the larger deviation from 100% in the total Faradaic balance for the Co-doped In_2_O_3_ is caused by an underestimation of the hydrogen concentration in the exit gas stream due to the high quantities of hydrogen exceeding the linear region of hydrogen detection in the GC setup).

The GDEs containing the In_2_O_3_-based electrocatalysts, carbon and PTFE were evaluated for their catalytic performance in the CO_2_ER in a flow cell. The measurements were carried out at (geometrical) current densities of 100, 150, and 200 mA/cm^2^ to compare the catalyst at industrially relevant conditions. In a previous study, the electrode configuration employed here was optimized to avoid mass transport limitations and ensure sufficient catalytic surface area.^55^ We earlier showed that, above a catalyst loading of 0.17 mg/cm^2^, a current density of 200 mA/cm^2^ can be obtained without affecting the Faradaic efficiency toward formate (FE_formate_). Here, using an electrode with a catalyst loading of 0.5 mg/cm^2^, we could reach a current density of up to 300 mA/cm^2^ while maintaining a constant FE_formate_ (Figure S7). This indicates that CO_2_ transport is not limiting the FE_formate_ at the employed current densities. The main products observed here were formate, CO, H_2_, and hydrocarbons. The FE of hydrocarbons, mainly CH_4_, was always less than 1%. Figure 2 shows the FE toward these products as a function of the current density. Figure S6 shows the potentials required to maintain these current densities. At current densities of 100 and 150 mA/cm^2^, the FE_formate_ reaches ∼86% for In_2_O_3_ and the Zr- and Ce-doped In_2_O_3_. For Pd- and Ni-doped In_2_O_3_, the FE_formate_ is slightly lower, namely at 79% and 74%, respectively, at these current densities. The FE_formate_ for Co-doped In_2_O_3_ is very low at 37% due to the much higher FE to H_2_. Increasing the current density to 200 mA/cm^2^ results in a minor but significant difference in FE_formate_ between Zr- and Ce-doped In_2_O_3_ (85 and 86%, respectively) compared to In_2_O_3_ (81%). The presence of Pd shows, at all current densities, the highest FE to CO, which is most likely due to the formation of metallic Pd or Pd-hydrides, which are active in CO_2_ reduction to CO instead of formate.^56,57^ The data show a trend of increasing FE toward H_2_ and CO when a lower potential is applied to maintain a given current density (Figure S6). This can be attributed to a lower overpotential of the competing HER and CO_2_ to CO reduction reaction for the samples that contain Pd, Ni, and Co.

### Surface Characterization of Ce- and Co-Doped In_2_O_3_

Based on the galvanostatic measurements at a current density of 200 mA/cm^2^, the FE_formate_ decreases in the order Ce-doped In_2_O_3_ > Zr-doped In_2_O_3_ > In_2_O_3_ > Pd-doped In_2_O_3_ > Ni-doped In_2_O_3_ > Co-doped In_2_O_3_. This order trends with the minor shift in the binding energy of the main In^3+^ 3d_5/2_ state, suggesting that the impact of dopants on the electronic state of In cations at the surface correlates with the CO_2_ER to formate. Care must be taken, however, as some of the dopants can be active for electrochemical CO_2_ reduction, especially for the HER. Moreover, some of the compositions might reduce to the metallic form during the CO_2_ER. To study this aspect further, we characterized three samples in more detail by cyclic voltammetry (CV).

**Figure 3:**
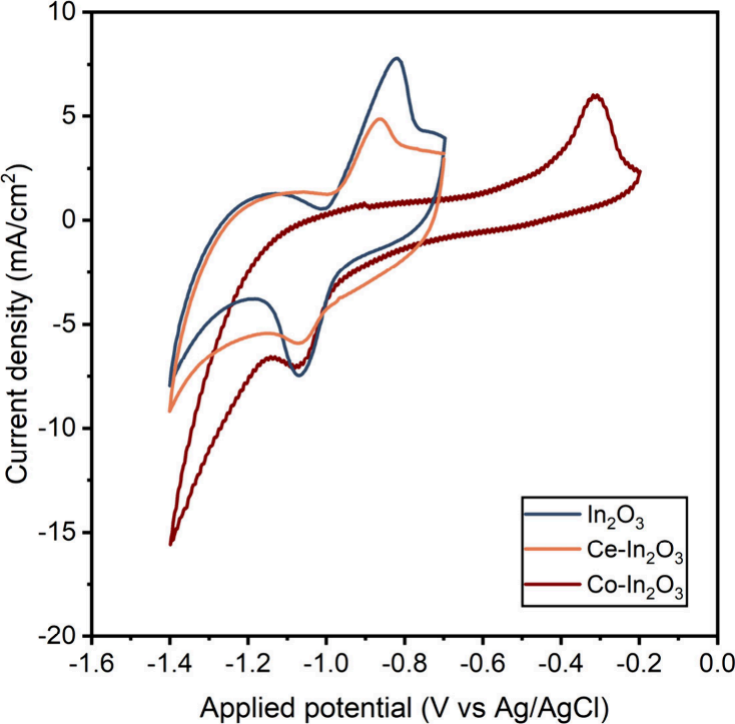
Cyclic voltammetry in 0.5 M KCHO_3_ saturated with CO_2_, on GDEs with In_2_O_3_ (0.35 mg/cm^2^, blue line), Ce–In_2_O_3_ (0.46 mg/cm^2^, orange line), and Co–In_2_O_3_ (0.27 mg/cm^2^, red line). Scan rate: 100 mV/s.

Figure 3 compares the CVs of In_2_O_3_ doped with Ce and Co with the one of In_2_O_3_. The CV of In_2_O_3_ contains a cathodic peak at −1.0 V vs Ag/AgCl, which corresponds to the reduction of In^3+^ to metallic In, and an anodic peak at −0.9 V vs Ag/AgCl due to reoxidation of metallic In to In^3+^.^21^ As such, these potentials represent the reversible character of the In^3+^/In^0^ redox couple in this sample. The oxidation peak in Co–In_2_O_3_ is shifted to a significantly higher anodic potential of −0.4 V vs Ag/AgCl, while the reduction is hardly affected. Based on the observation of the identical location of the reduction peak, and the absence of other peaks, we speculate that the CV still represents the In^3+^/In^0^ redox couple and that doping Co in In_2_O_3_ makes the In^3+^/In^0^ redox couple significantly less reversible. This is attributed to Co doping of the metallic In phase obtained upon reduction. This results in a higher barrier for oxidation of Co-doped In compared to monometallic In. We explain this by the formation of a Co–In alloy for which the In oxidation potential shifts closer to the oxidation potential of Co metal to Co^2+^.^58^ On the other hand, XPS analysis before and after CO_2_ER reveals a slight increase in Co-concentration from 12 to 15 at% (Figure S8d). This can indicate that Co migrates to the surface of the particle, which can explain the shift of the oxidation peak closer to that of pure Co. As such, the formation of a monolayer of Co cannot be fully excluded. Doping with Ce does not affect the oxidation and reduction potentials of In_2_O_3_.

**Figure 4:**
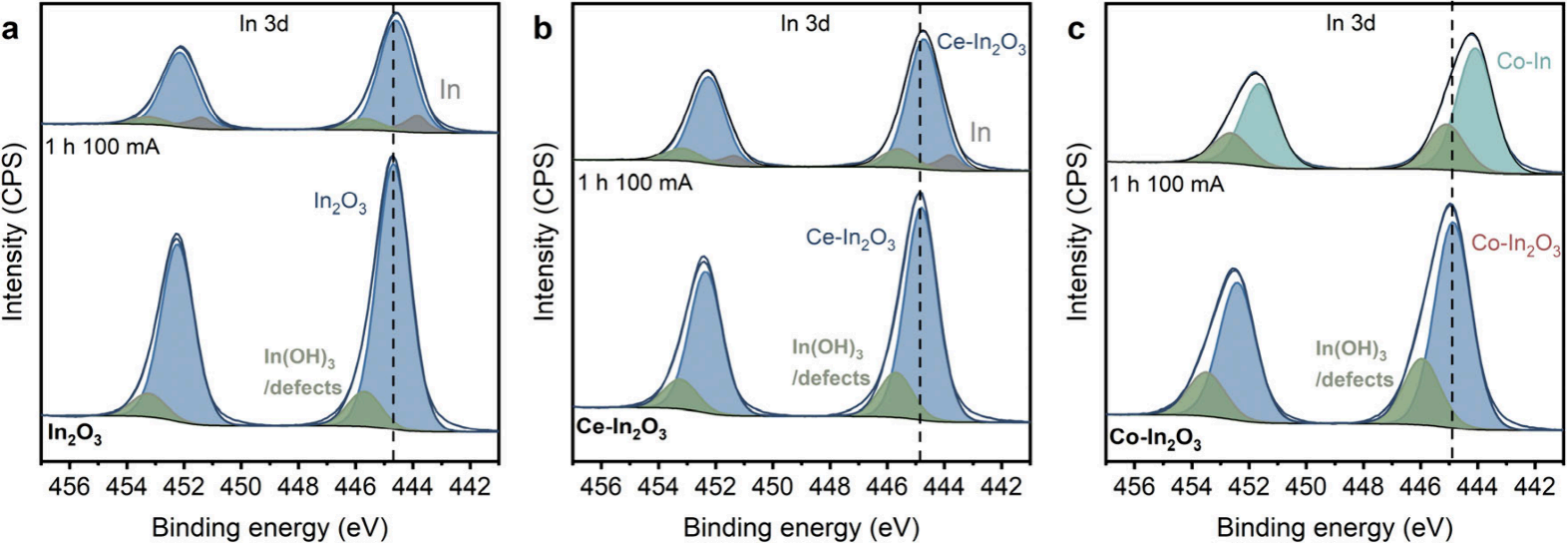
XPS analysis of the In 3d core-line region before and after CO_2_ER using a GDE with pure In_2_O_3_ (a, blue), Ce-doped In_2_O_3_ (b, orange), and Co-doped In_2_O_3_ (c, red) GDEs. After electrochemical CO_2_ reduction, the electrode is removed from the electrochemical flow cell and transferred through air to the XPS apparatus.

XPS before and after CO_2_ER was used to analyze possible changes in the surface composition. The electrocatalysts used for CO_2_ER were transferred through air to the XPS apparatus, which might lead to oxidation of the surface. Figure 4 shows the In 3d spectra for the fresh and used In_2_O_3_, Ce–In_2_O_3_, and Co–In_2_O_3_ GDEs, which were used to determine the surface composition of the samples (Table S1). The surface concentration of Ce decreased from 10.1 to 5.6 at% after 1 h CO_2_ER, indicating that there was some migration of In to the surface of the nanoparticles during the electrochemical reaction or dissolution of Ce from the surface. On the contrary, the surface concentration of Co increased from 12 to 15 at% (Figure S8c, d). The enrichment in Co of the surface might indicate the formation of a Co-rich alloy close to the surface during CO_2_ER.

The spectra were deconvoluted to determine possible changes in the oxidation state (Figure 4). The In XPS spectra of the used samples contain an additional metallic contribution at an In 3d_5/2_ binding energy of 443.8 eV (Figure S8a). The In^0^ contribution is slightly lower (7.2 at%) for used Ce–In_2_O_3_ than for used In_2_O_3_ (10.6 at%) as shown in Table S1. The In 3d spectrum of used Co–In_2_O_3_ sample differs the most from the fresh sample among the set. The contribution of the In^3+^ due to In_2_O_3_ has significantly decreased, while the main feature is at 444.1 eV. Most likely, this reflects the reduction of a significant fraction of In to metallic In. The higher binding energy compared to metallic In can be due to the doping with the slightly more electronegative Co in the lattice. XRD analysis of the GDE after CO_2_ER hints at the formation of some metallic In (Figure S8b). The diffractogram does not provide enough resolution to conclude on the presence of In_2_O_3_ or a CoIn_3_ alloy, which is due to overlap of peaks from abundantly present PTFE.^59^

XPS indicates that the oxidation state of the In_2_O_3_ phase is influenced by the dopants during CO_2_ER. Ce might inhibit the reduction of the In_2_O_3_ phase as observed from the lower metal In content after CO_2_ER, while In_2_O_3_ reduction is promoted by Co. Metallic Co could be an active catalyst for HER.^60,61^ The relatively large surface contribution of Co on the surface, possibly forming a Co–In alloy, most likely contributes to the increased FE toward H_2_ observed in Figure 2. However, the increased reduction of In_2_O_3_ to metallic In caused by Co could also negatively impact the adsorption of the oxygen-bound formate intermediate, decreasing the FE_formate_.^10^

### Ce–In Oxide Catalysts

To further investigate the effect of Ce dopant on In_2_O_3_ and its activity in CO_2_ER toward formate, a range of new catalysts were synthesized by FSP with Ce contents of 0, 10, 50, and 90 at%. The main physicochemical properties of the Ce–In_2_O_3_ catalysts are given in Table 2. The catalysts are denoted by their atomic Ce content, i.e., Ce(50)-In_2_O_3_ having a Ce content of roughly 50 at%.

**Figure 5:**
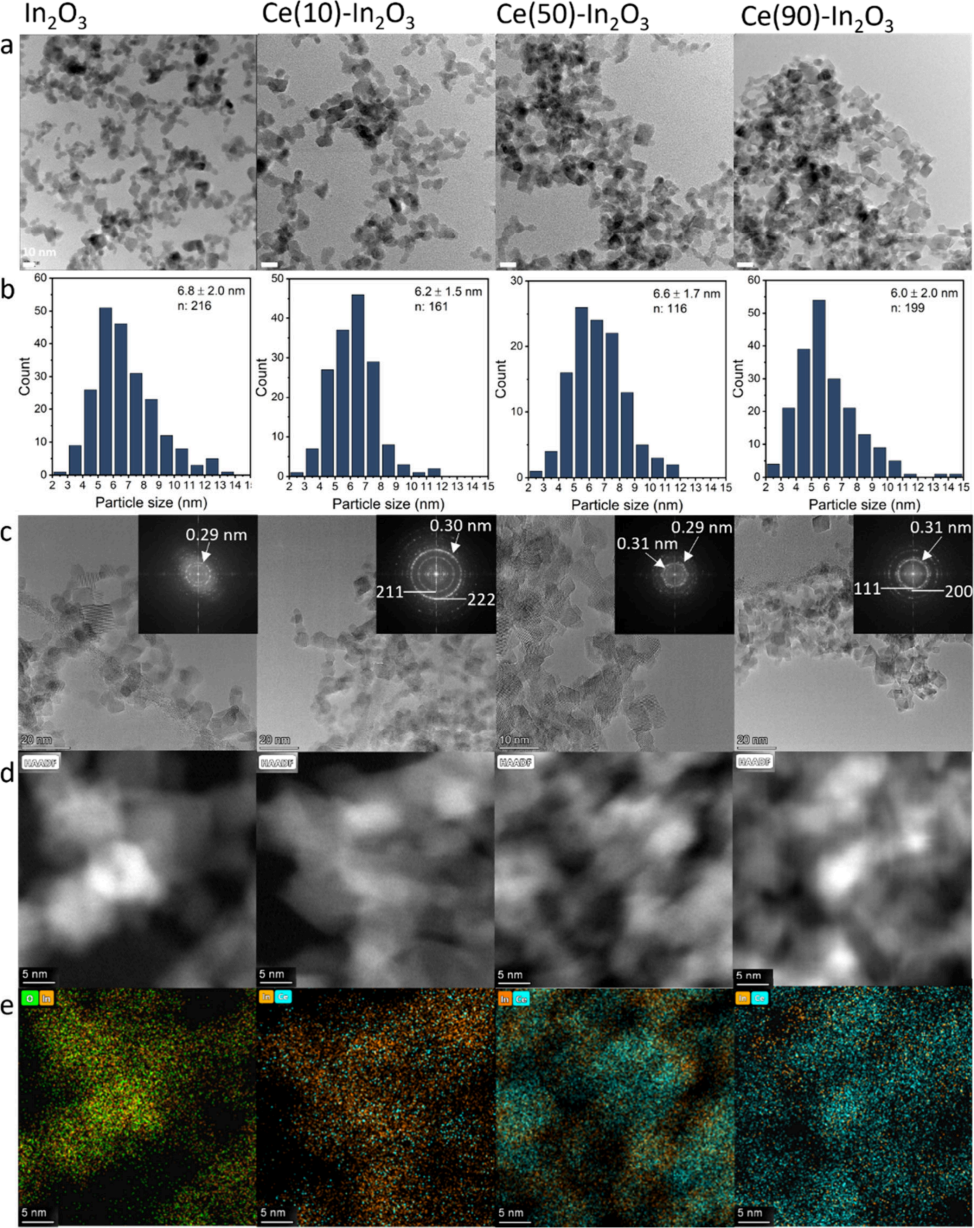
(a) TEM images of the FSP synthesized In_2_O_3_ and Ce–In_2_O_3_ catalysts with corresponding size distributions (b). (c) HRTEM images and FFT showing the high (mono-) crystallinity of the nanoparticles and lattice spacing. (d–e) STEM-EDX imaging of the particles showing the high dispersion of In and Ce atoms in the mixed catalysts.

TEM was used to determine the shape and size of the Ce–In_2_O_3_ nanoparticles, while STEM-EDX revealed the nanoscale distribution of Ce with respect to In. The TEM images and particle size distributions given in Figure 5a and 5b show that the Ce–In_2_O_3_ samples are predominantly made up of particles with a size of 6–7 nm as also observed for the other doped In_2_O_3_ catalysts. This shows that the particle size for these samples is hardly affected by the composition, with Ce-rich compositions giving nearly the same average particle size. The shape of the In_2_O_3_ particles is mostly spherical, although some cubes are also observed. An increase in the Ce content leads to the formation of octahedral particles, which is the dominant shape of CeO_2_ prepared by FSP.^62^ The TEM images in Figure 5 reveal the crystallinity of the particles. The lattice spacings as determined from the TEM images (Figure 5c and Figure S9) show a small increase in the In_2_O_3_ (222) lattice spacing from 0.29 nm for the In_2_O_3_ sample to 0.30 nm for the Ce(10)-In_2_O_3_ one. From the images of Ce(50)-In_2_O_3_, we can derive the presence of particles with lattice spacings of 0.29 and 0.30, which can be related to (222) planes of (Ce-doped) In_2_O_3_ as well as 0.31 nm, which corresponds to the (111) plane of CeO_2_. For Ce(90)-In_2_O_3_, the lattice spacing of 0.31 nm is most frequently observed, indicating the predominance of CeO_2_ in this sample. HAADF imaging in combination with EDX gives further insight into the dispersion of In and Ce in the particles (Figure 5d and 5e). Ce is well dispersed over the In_2_O_3_ particles in Ce(10)-In_2_O_3_. For the Ce(50)-In_2_O_3_ sample, different phases can be observed with smaller particles predominantly containing In surrounding larger particles rich in Ce. The Ce(90)-In_2_O_3_ appears to consist of CeO_2_ particles, in which In is present at high dispersion next to some regions with more clustered forms of In-oxide.

**Figure 6:**
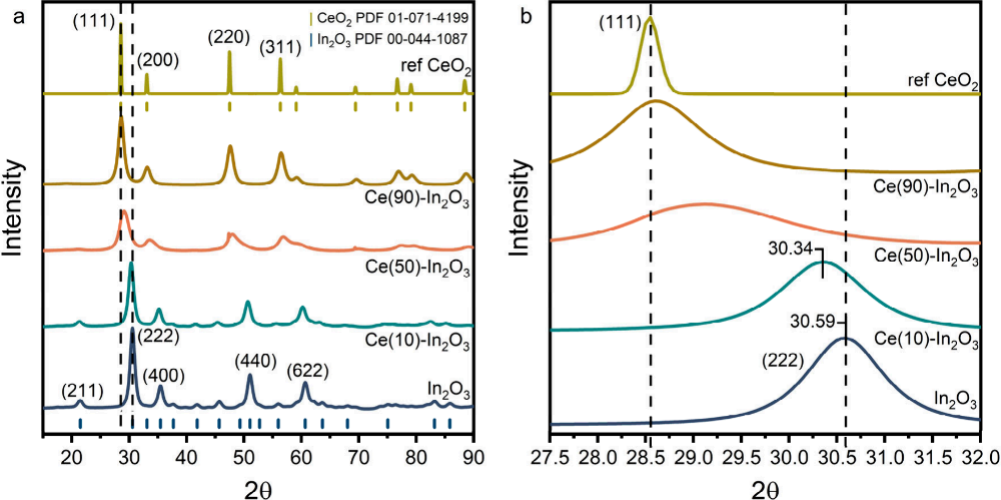
(a) sXRD patterns of Ce–In_2_O_3_ catalysts synthesized by FSP. In_2_O_3_ and CeO_2_ phases are characterized according to PDF cards 00-044-1087 and 01-071-4199 respectively. Besides In_2_O_3_, Ce(10)-In_2_O_3_, Ce(50)-In_2_O_3_, and Ce(90)-In_2_O_3_, a reference sample of CeO_2_ (top) is analyzed. (b) Zoom in on the In_2_O_3_ (222) and CeO_2_ (111) diffraction lines showing the shifts indicative of lattice expansion and contraction by Ce- and In-dopants, respectively.

Synchrotron-based XRD (sXRD) was employed to study the phases present in the Ce–In_2_O_3_ samples in more detail (Figure 6). Qualitatively, the patterns suggest that Ce(10)-In_2_O_3_ is mainly composed of In_2_O_3_ in which Ce was doped, while Ce(50)-In_2_O_3_ and Ce(90)-In_2_O_3_ are predominantly made up of CeO_2_ in which In was doped. The (222) and (111) diffraction lines of respectively In_2_O_3_ and CeO_2_ were fitted by a Voigt function to estimate the crystallite size using the Scherrer equation. The results are listed in Table 2. The crystallite size of In_2_O_3_ of 8.6 nm determined by sXRD is in good agreement with the value of 9.2 nm determined by lab-based XRD (Table 1). The same holds for the crystallite size of Ce(10)-In_2_O_3_ determined by sXRD (8.2 nm) and XRD (7.8 nm). Based on Rietveld refinement of the sXRD patterns, there are no signs of CeO_2_ admixtures in the Ce(10)-In_2_O_3_ sample, indicating that possible CeO_2_ admixtures should either be very small or absent (Figure S12). The (222) diffraction line of pure In_2_O_3_ is located at 30.59 degrees, corresponding to a lattice spacing of 2.92 Å. A small shift to lower diffraction angles for Ce(10)-In_2_O_3_ indicates an increase in the lattice parameter. The In_2_O_3_ lattice parameter was determined by Rietveld refinement at 10.19 Å, corresponding to an increase of 0.05 Å compared to pure In_2_O_3_, which confirms the incorporation of Ce in the In_2_O_3_ lattice (Figure 6b and Figure S12). The sXRD pattern of Ce(90)-In_2_O_3_ contains a dominant (111) diffraction line representative of the CeO_2_ fluorite phase. The small shift to higher diffraction angle indicates inclusion of In in the CeO_2_ lattice. Scherrer analysis shows that the average size of the CeO_2_ particles is 7.1 nm. No In_2_O_3_ phase could be fitted using Rietveld refinement, indicating that possible In_2_O_3_ admixtures should either be very small or absent. The CeO_2_ lattice parameter was determined at 5.40 Å (Figure S12). The minor decrease (0.01 Å) compared to pure CeO_2_ confirms the inclusion of some In in the CeO_2_ lattice. The Ce(50)-In_2_O_3_ appears to consist of a mixture of CeO_2_, which contains more doped In than Ce(90)-In_2_O_3_, as indicated by the relatively large shift of the CeO_2_ diffraction lines, and Ce-doped In_2_O_3_. The presence of Ce-doped In_2_O_3_ can be deduced from the shoulder at the (220) peak of CeO_2_. Further Rietveld refinement confirms the presence of ∼50 wt % of both phases. The lattice parameters of the In_2_O_3_ and CeO_2_ phases are respectively 10.43 and 5.37 Å. The changes in lattice parameter confirm the inclusion of Ce in the In_2_O_3_ phase and In in the CeO_2_ phase (Figure S12). Scherrer analysis reveals that the CeO_2_ particles in this sample are much smaller at 4.6 nm than in the other samples. Comparing these findings to the STEM-EDX images in Figure 5e, it can be said that the Ce(50)-In_2_O_3_ consists of small 5 nm sized CeO_2_ particles doped with In, which support small In_2_O_3_ particles doped with Ce.

The surface composition of the Ce–In_2_O_3_ catalysts was analyzed by XPS and listed in Table 2. The surface Ce contents are 8.0, 38, and 83 at% for respectively Ce(10)-In_2_O_3_, Ce(50)-In_2_O_3_, and Ce(90)-In_2_O_3_. The slightly lower Ce contents compared to the nominal values of 11, 51.5, and 92.6 at% hint at surface enrichment of In as also found for the Ce–In_2_O_3_ sample in the initial set. Qualitatively, this is also in line with the STEM-EDX images for Ce(50)-In_2_O_3_, showing small In_2_O_3_ surrounding larger CeO_2_ particles (Figure 5e). The metal distribution in the other samples seems to be more homogeneous (Figure S10 and Figure S13).

**Figure 7:**
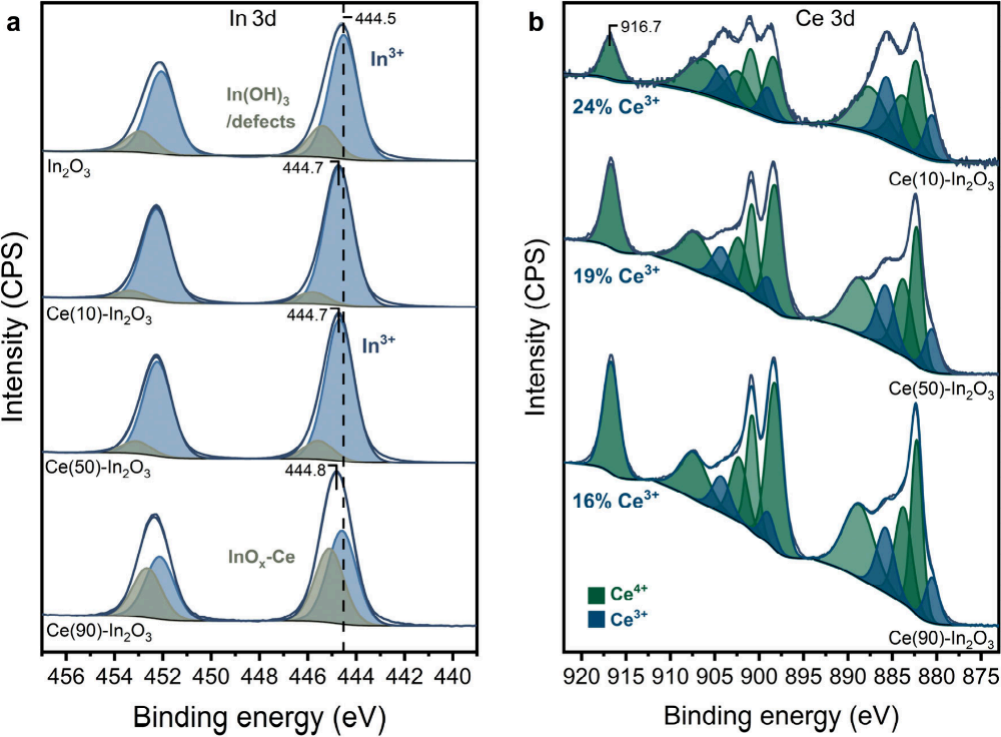
(a) XPS analysis of the In 3d core-line region of pelletized catalyst particles of In_2_O_3_, Ce(10)-In_2_O_3_, Ce(50)-In_2_O_3_, and Ce(90)-In_2_O_3_. (b) XPS analysis of the Ce 3d core-line regions of the Ce–In catalysts. The spectra were deconvoluted into features belonging to Ce^3+^ and Ce^4+^ oxidation states in the same way as reported by Muravev et al.^33^ Ce^3+^ and Ce^4+^ related features are shown in blue and green, respectively. All spectra were energy corrected by the U‴ component at 916.7 eV of Ce.^31^ The resulting C 1s binding energy (measured at 285.3 eV) was used to energy correct the spectrum of pure In_2_O_3_.

The XPS spectra of the In 3d region given in Figure 7a were fitted in the same way as described above. This results in two contributions, a main feature due to In_2_O_3_ at an In 3d_5/2_ binding energy of 444.5 eV and a small one at a higher binding energy due to In(OH)_3_/In_2_O_3_-defects. Figure 7a shows the small shift in the binding energy of 0.2 eV of the main In 3d_5/2_ peak in Ce(10)-In_2_O_3_, as was also observed for Ce-doped In_2_O_3_ in Figure 1. The binding energy and the shift compared to In_2_O_3_ do not change significantly for the Ce(50)-In_2_O_3_ and Ce(90)-In_2_O_3_ samples. The contribution of In(OH)_3_/In_2_O_3_-defects increases, however, with increasing Ce content. Especially, the Ce(90)-In_2_O_3_ contains a large contribution of 45% of such states (Table 2), which can be attributed to the high dispersion of In-oxide in this sample in line with the high In dispersion observed by STEM-EDX (Figure 5e).

Figure 7b shows the Ce 3d XPS spectra of these samples including the Ce^3+^ and Ce^4+^ contributions. The Ce^3+^ content is lowest for Ce(90)-In_2_O_3_ (16 at%), These Ce^3+^ ions reflect oxygen vacancies in the CeO_2_ lattice.^45^ The Ce^3+^ content increases to 19 at% in Ce(50)-In_2_O_3_ and 24 at% in Ce(10)-In_2_O_3_, the latter value being similar to the one for Ce-doped In_2_O_3_ in Figure S5a. The higher Ce^3+^ content at a lower nominal Ce content is most likely due to the smaller size of the CeO_2_ particles. At the lowest Ce content (highest In content), it can also be that the 3+ oxidation state is preferred for Ce, substituting In. We note that Ce_2_O_3_ occurs in the same cubic crystal structure as In_2_O_3_ with the *Ia3* space group with a Ce–O bond distance of 2.4 Å (In–O bond distance is 2.2 Å), but no Ce_2_O_3_ phase was found from Rietveld refinement.^45,63,64^

**Figure 8:**
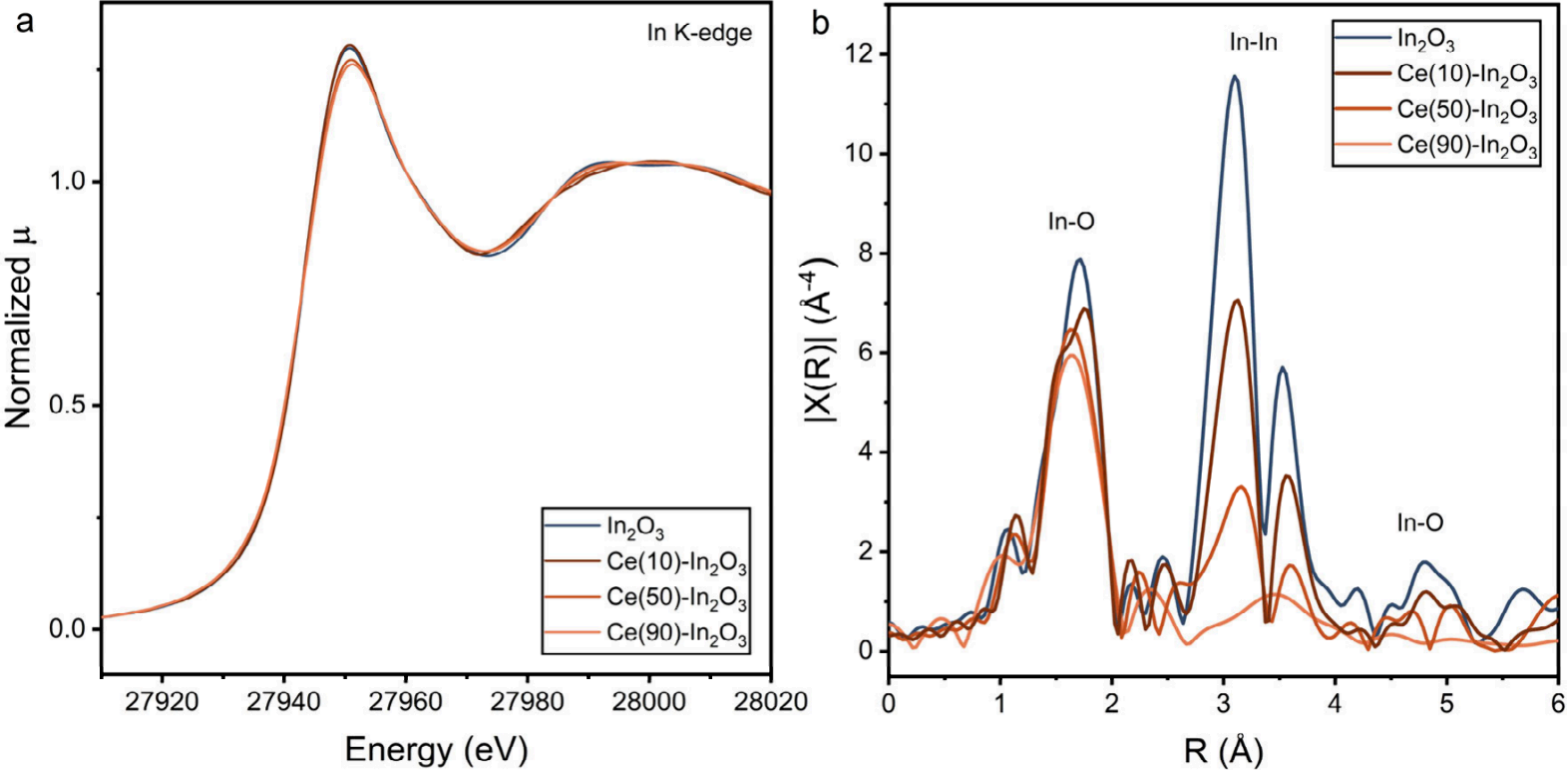
(a) In K-edge XANES of for In_2_O_3_, Ce(10)-In_2_O_3_, Ce(50)-In_2_O_3_, and Ce(90)-In_2_O_3_ before CO_2_ER. (b) The k^3^ weighted FT-EXAFS spectra (In K-edge) of In_2_O_3_ and Ce–In_2_O_3_ catalysts before CO_2_ER.

X-ray absorption near edge spectroscopy (XANES) spectra at the In K-edge of the In_2_O_3_ and Ce–In_2_O_3_ samples are shown in Figure 8a. All XANES spectra are very similar to those of In_2_O_3_, reflecting the predominant 3+ oxidation state of In. The XANES spectra of reference In metal is plotted in Figure S15, showing the lower edge energy of metallic In. In_2_O_3_ forms a cubic crystal structure of the *Ia3* space group and contains two different In^3+^ sites, forming (distorted) InO_6_ octahedra with In–O bonds around 2.2 Å.^65^ The extended X-ray absorption fine structures (EXAFS) in Figure 8b show an intense first In–O coordination shell. The second shell corresponds to an In–O–In coordination shell with two different bond distances between the In atoms in the cubic In_2_O_3_ crystal structure.^65−68^ This second shell decreases with increasing Ce content, which can indicate a decrease of the size of the In_2_O_3_ particles or the replacement of In in the second shell by Ce. A weak second shell remains for the Ce(90)-In_2_O_3_ sample. Fits of these EXAFS are given in Figure S16. The fit parameters in Table S2 reveal a decrease in the second shell ln–O-In coordination number from 11.8 ± 1.7 for In_2_O_3_ to 4.1 ± 3.0 in Ce(90)-In_2_O_3_ (Table S2). This result is qualitatively in agreement with the findings from STEM-EDX, XPS and sXRD that Ce(90)-In_2_O_3_ contains small In-oxide particles covering CeO_2_.

**Figure 9:**
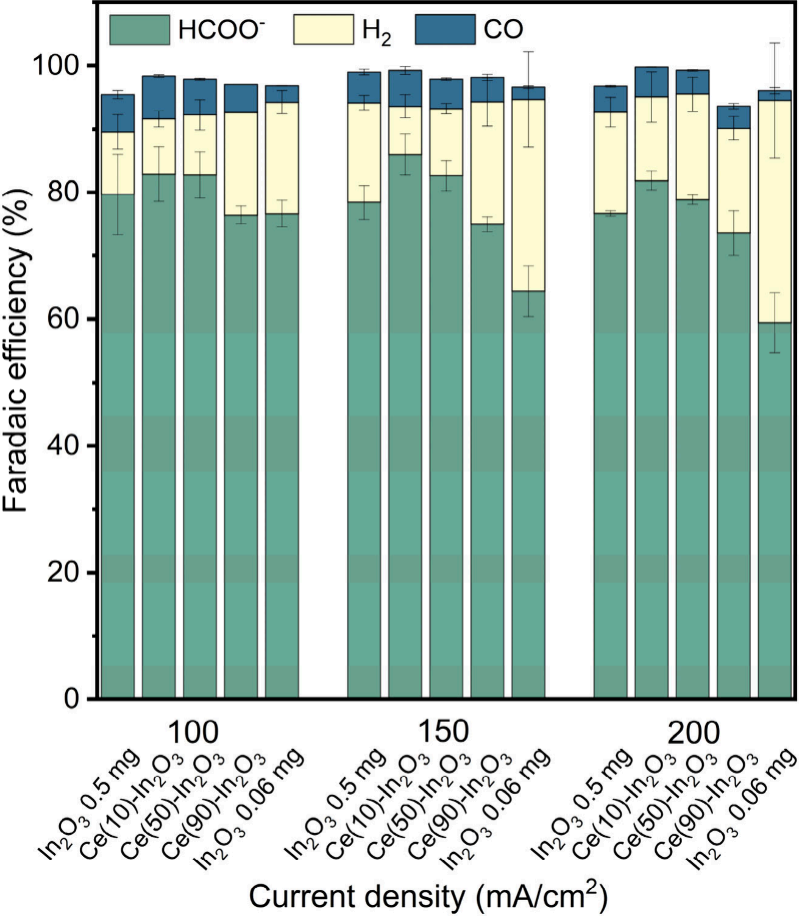
Faradaic efficiency as a function of current density on In_2_O_3_ and Ce–In_2_O_3_ catalysts after 1 h CO_2_ER in 0.5 M KHCO_3_. Catalyst nanoparticles are deposited on a GDE (0.47 ± 0.07 mg/cm^2^). Experiments were repeated in duplicate to obtain the error bars.

The catalytic performance of the Ce–In_2_O_3_ samples was evaluated in the same way as discussed above. The FE_formate_ as a function of the current density are given in Figure 9. In line with the result in Figure 2, the Ce(10)-In_2_O_3_ sample exhibits an increased FE_formate_ compared to In_2_O_3_ at current densities of 150 and 200 mA/cm^2^. The maximum FE_formate_ for this sample is 86 ± 3% at 150 mA/cm^2^, which compares favorably to 78 ± 3% for In_2_O_3_. The higher Ce content in Ce(50)-In_2_O_3_ results in a maximum FE_formate_ of 83 ± 2% at a current density of 150 mA/cm^2^. At the highest Ce content in Ce(90)-In_2_O_3_, the FE_formate_ decreases slightly to 75% and 74% at respective current densities of 150 and 200 mA/cm^2^, which is below the performance of In_2_O_3_. It should be noted that these values are obtained with a relatively low In content in the GDE (∼0.05 mg In_2_O_3_/cm^2^). This is below the minimum necessary catalyst loading observed in our previous study with this electrode configuration.^55^ To determine whether CeO_2_ plays a synergistic role with respect to In_2_O_3_, we determined the performance of In_2_O_3_ at a loading of the GDE of 0.06 mg/cm^2^, which is nearly the same as the In_2_O_3_ loading used for the Ce(90)-In_2_O_3_ (∼0.05 mg/cm^2^). The results in Figure 9 for current densities above 100 mA/cm^2^ clearly show that CeO_2_ has a positive influence on In_2_O_3_ by the substantially higher FE_formate_ for the Ce(90)-In_2_O_3_. The lower FE_formate_ for the GDE with an In_2_O_3_ loading 0.06 mg/cm^2^ stems from the much larger contribution of the HER. The catalytic activity of CeO_2_ as shown in Figure S11 demonstrates that CeO_2_ is a poor electrocatalyst for CO_2_ reduction, reaching a FE_formate_ of 17% at 100 mA/cm^2^. The FE_formate_ decreases to 8% at 200 mA/cm^2^. HER is the main reaction here competing with formate production in addition to the formation of a small amount of CO. On the Pd-, Ni-, and Co-doped In_2_O_3_ samples, the FE_formate_ and average applied potential decreased compared to on In_2_O_3_ (Figure S6), most likely due to a lower kinetic barrier for hydrogen evolution. The applied potential on Ce(10)-In_2_O_3_ and Ce(50)-In_2_O_3_ are respectively lower and similar compared to on In_2_O_3_ (Figure S14). Nevertheless, the higher FE_formate_ points to a lower kinetic barrier for CO_2_ reduction to formate. On Ce(90)-In_2_O_3_ and CeO_2_, the applied potential increases, while the FE_formate_ decreases compared to on In_2_O_3_. This is most likely, because pure CeO_2_ is a poor electrocatalyst for both hydrogen evolution and CO_2_ reduction and cannot maintain a high current density without sufficient In. To understand the changes in intrinsic catalytic activity of the Ce-doped or Ce-supported In_2_O_3_, such as the onset potential for CO_2_ER, potentiostatic measurements on a rotating disk electrode could give better control over the applied potential and more insight in the overpotential of the catalysts toward CO_2_ electroreduction.

The increase in FE brought about by the Ce in Ce(10)-In_2_O_3_ could be caused by a change in the electronic structure of In_2_O_3_. The higher binding energy of the valence electrons on the In atom might stabilize the In_2_O_3_ phase. To understand the contribution of Ce to the electronic structure of In_2_O_3_, the catalysts were further analyzed using operando XAS.

### Structural Evolution of Ce–In Catalysts during CO_2_ER

**Figure 10:**
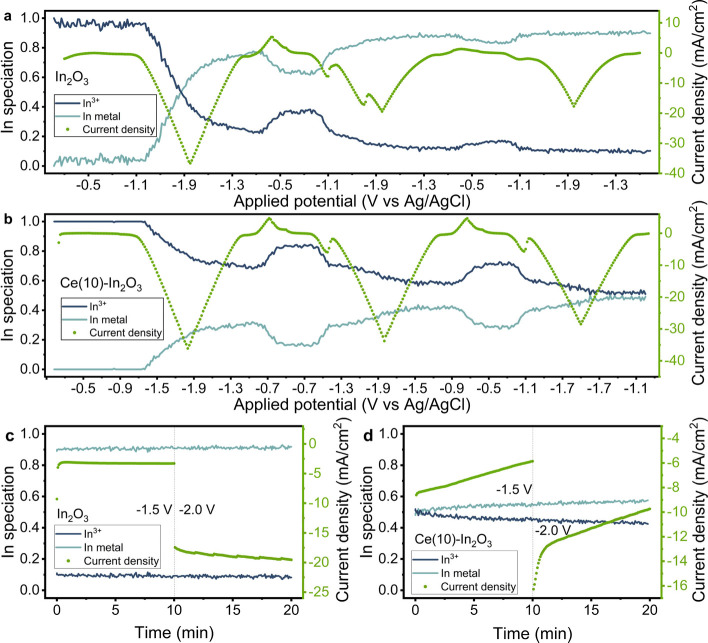
Potential resolved evolution of In speciation determined by linear combination analysis of XAS spectra for In_2_O_3_ (a) and Ce(10)-In_2_O_3_ (b) during cyclic voltammetry in 0.5 M KHCO_3_ at a scan rate of 10 mV/s. The potential is displayed on the *X*-axis in chronological order, sweeping back and forth between −0.4 V and −2 V vs Ag/AgCl. The green scattered plot gives the recorded current density, reaching a maximum of −2.0 V vs Ag/AgCl at every cycle. (c, d) Time-resolved evolution of In speciation determined by linear combination analysis of XAS spectra for In_2_O_3_ (c) and Ce(10)-In_2_O_3_ (d) during chrono-amperometry at −1.5 V and −2.0 V vs Ag/AgCl after 3 CVs.

Using operando XANES at the In K-edge, the chemical state of In was characterized during cyclic voltammetry and chronoamperometry on a modified GDE during CO_2_ reduction. The temporal resolution of the XANES measurements (0.5 s/scan) was used to follow the spectral changes at the In K-edge during cyclic voltammetry. The potential on the electrode was cycled between −0.4 V and −2.0 V vs Ag/AgCl for 3 CVs before CO_2_ reduction was followed at constant potentials of −1.5 and −2.0 V vs Ag/AgCl. Figure S17 shows the corresponding CVs for the In_2_O_3_ and Ce–In_2_O_3_ samples. During the first cathodic scan for In_2_O_3_ (Figure S17a), the current density increases from the onset potential of −1.1 V vs Ag/AgCl onward until the most negative applied potential of −2.0 V vs Ag/AgCl. Linear combination fitting (LCF) reveals the changes in the In oxidation state during CV cycling (Figure 10a). At −1.1 V vs Ag/AgCl, the increase in current coincides with rapid reduction of In_2_O_3_ to metallic In, obtaining almost 80 at% metallic In after completion of the cathodic cycle. XANES spectra at −1.0 V, −1.5 V, −2.0 V vs Ag/AgCl and EXAFS spectra during the first cathodic cycle show that In_2_O_3_ reduction continues during the cathodic cycle until the potential and current return to the nonfaradaic region in the CV (Figure S18). During the anodic cycle, a minor reoxidation feature of metallic In starting at −0.9 V vs Ag/AgCl can be observed, which results in the presence of ca. 35 at% In_2_O_3_. During the second cycle, the maximum cathodic current density is lower than in the first cycle, and two cathodic peaks are observed. The first cathodic peak starting at −0.9 V vs Ag/AgCl coincides with the reduction of reoxidized In_2_O_3_, yielding again a metallic In contribution of 80 at%. The second reduction feature starts around −1.4 V vs Ag/AgCl but does not lead to a significant change in the In speciation according to XANES. Most likely, this feature represents the reduction of CO_2_. In the third cycle, only minor reoxidation occurs. Reduction during the third cathodic scan results in a nearly constant In speciation of 90% metallic In. Subsequent CO_2_ reduction at constant potentials of −1.5 and −2.0 V vs Ag/AgCl does not further change the In speciation (Figure 10c). Contrary to observations in our previous work using in situ Raman spectroscopy,^55^ a small amount of In_2_O_3_ remains present up to −2.0 V vs Ag/AgCl. The presence of residual In_2_O_3_ may have a role in the CO_2_ reduction reaction, as it was argued to be a more active phase for CO_2_ reduction to formate.^17^ A remaining peak of the In–O shell in the EXAFS confirms the presence of In–O and/or In–OH (Figure S19b). Fitting of the first In–O shell shows that the In–O coordination number decreased to 0.8 ± 0.5 and the In–O bond distance decreased from 2.2 to 2.0 Å. All this suggests a predominantly metallic In phase with some residual In–O bonds.

The reduction of In_2_O_3_ is slower in the presence of Ce as can be judged from the CV-XANES experiment for Ce(10)-In_2_O_3_. After the first CV, the metallic In content is only 30 at% (Figure 10b). During the subsequent 2 cycles, more In is reduced, attaining a metallic In content of 50 at%, significantly less than the nearly full reduction (90 at%) obtained for In_2_O_3_. During chronoamperometry at −1.5 V and −2.0 V vs Ag/AgCl, the reduction of In_2_O_3_ to metallic In continues, reaching 60% metallic In at −2.0 vs Ag/AgCl after 20 min (Figure 10d).

**Figure 11:**
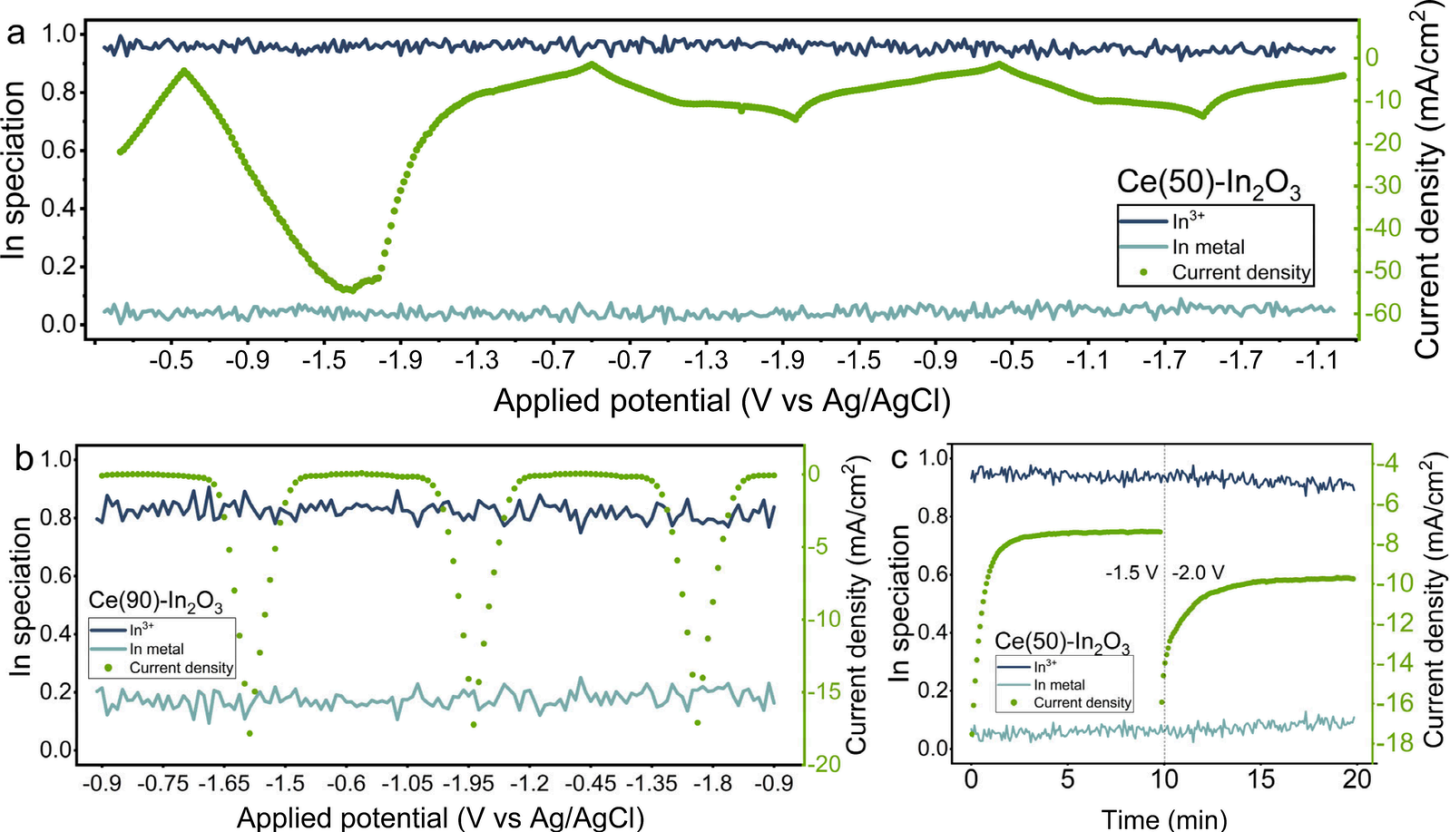
Potential and time-resolved evolution of In speciation determined by linear combination analysis of XAS spectra for Ce(50)-In_2_O_3_ (a) and Ce(90)-In_2_O_3_ (b) during cyclic voltammetry in 0.5 M KHCO_3_ at a scan rate of 10 mV/s. (c) Ce(50)-In_2_O_3_ catalyst during chrono-amperometry at −1.5 V and −2.0 V vs Ag/AgCl after 3 CVs.

At a higher Ce content of 50 at%, the reduction of In_2_O_3_ to metallic In is strongly suppressed (Figure 11a). The large cathodic current observed for the Ce(50)-In_2_O_3_ sample during the first CV (Figure S17c) could be due to the reduction of CO_2_, CeO_2_,^100,101^ or any contamination in the experiment. No further reduction was observed during chronoamperometry at −1.5 V vs Ag/AgCl. Nevertheless, at a constant potential of −2.0 V vs Ag/AgCl, a slight reduction of In_2_O_3_ to metallic In was seen (Figure 11c). Suppression of In_2_O_3_ reduction by Ce is also evident for the Ce(90)-In_2_O_3_ sample, despite the lower signal-to-noise ratio of the XANES spectra (Figure 11b and Figure S20). The low quality of the spectra due to the low In content hampered meaningful LCF. Reoxidation of In was followed at open circuit potential (OCP) after the CO_2_ER in all experiments (Figure S21). A slight reoxidation of In to In_2_O_3_ was observed for all catalysts. The In_2_O_3_ fraction was found to increase 2% for In_2_O_3_ and 8% for Ce(10)-In_2_O_3_ after 5 min.

The suppression of In_2_O_3_ reduction correlates well with the observed decreased efficiency toward H_2_ and increased efficiency toward CO_2_ electroreduction. Potentiostatic analysis is needed to provide more information for the change in onset potential toward H_2_O reduction and CO_2_ reduction. Besides a high current density, a stable catalyst is also required for industrial applicability. This study is limited to measurements of 1 h to identify the effect of In_2_O_3_ promotion. If Ce leaches out from the catalyst, as might be suggested by XPS analysis, stabilization of the Ce–In mixed phase could be explored by adding a structural promotor, as was demonstrated, for example, for In-sulfides by the group of Gao.^69^

## Conclusions

In_2_O_3_ nanoparticles were doped with Pd, Co, Ni, Zr, and Ce using flame spray pyrolysis. XRD and XPS show a high dispersion of the dopants in In_2_O_3_ in addition to some segregated dopant oxide phases on In_2_O_3_. Galvanostatic measurements at a current density of 200 mA/cm^2^ show a decreasing FE_formate_ in the order Ce-doped In_2_O_3_ > Zr-doped In_2_O_3_ > In_2_O_3_ > Pd-doped In_2_O_3_ > Ni-doped In_2_O_3_ > Co-doped In_2_O_3_. XPS analysis shows that an increasing FE_formate_ goes along with increasing binding energy of the In atoms, suggesting that the dopants can slightly modify the surface properties of the In_2_O_3_. Overall, the highest FE_formate_ is obtained with Ce-doped In_2_O_3_ (86%), which compared favorably with the FE_formate_ of 81% for In_2_O_3_. The lowest FE_formate_ of 37% is observed at the other extreme for Co-doped In_2_O_3_. Further characterization indicates stabilization of a metallic Co–In alloy in the latter catalyst, which can explain the significant contribution of the HER. Overall, only Ce and Zr doping of In_2_O_3_ improve FE_formate_. The Ce content of Ce-doped In_2_O_3_ nanoparticles was then varied at 10, 50, and 90 at% by FSP. The resulting materials are typically In_2_O_3_ with Ce dopants and CeO_2_ clusters at low Ce content, while CeO_2_ with In dopants and In_2_O_3_ clusters make up the catalyst at Ce contents of 50 and 90 at%. Compared to In_2_O_3_, FE_formate_ increases for samples that contain 10 and 50 at% Ce and decreases slightly at 90 at% Ce. The highest FE_formate_ of 86 ± 3% is obtained for Ce(10)-In_2_O_3_ at a current density of 150 mA/cm^2^, significantly higher than the value of 78 ± 3% for In_2_O_3_. In situ XAS analysis shows that almost all In_2_O_3_ reduces to metallic In during cyclic voltammetry and CO_2_ER at −2.0 V vs Ag/AgCl. At a Ce content of 10 at%, the degree of In reduction is much lower at 60%, while a further increase to 50 and 90 at% almost completely suppresses In_2_O_3_ reduction. The improved FE_formate_ at a low dopant level is attributed to the stabilization of In_2_O_3_, which presumably has a lower activity in the competing HER reaction than metallic In. A too-high Ce content decreases the FE_formate_ due to the formation of CeO_2_, which is a poor catalyst for the CO_2_ER to formate.

## Supporting Information Available

Additional tables and figures, containing experimental data, material characterization, and images referred to in this article (DOCX)

## References

[ref1] De Luna P.; Hahn C.; Higgins D.; Jaffer S. A.; Jaramillo T. F.; Sargent E. H. What Would It Take for Renewably Powered Electrosynthesis to Displace Petrochemical Processes?. Science 2019, 364 (6438), aav3506 10.1126/science.aav3506. 31023896

[ref2] Vayenas C. G., White R. E., Gamboa-Aldeco M. E. Modern Aspects of Electrochemistry; Springer New York: New York, NY, 2008; Vol. 42.

[ref3] Schuler E.; Morana M.; Ermolich P. A.; Lüschen K.; Greer A. J.; Taylor S. F. R.; Hardacre C.; Shiju N. R.; Gruter G.-J. M. Formate as a Key Intermediate in CO2 Utilization. Green Chem. 2022, 24 (21), 8227–8258. 10.1039/D2GC02220F.

[ref4] van Putten R.; Wissink T.; Swinkels T.; Pidko E. A. Fuelling the Hydrogen Economy: Scale-up of an Integrated Formic Acid-to-Power System. Int. J. Hydrogen Energy 2019, 44 (53), 28533–28541. 10.1016/j.ijhydene.2019.01.153.

[ref5] Han N.; Ding P.; He L.; Li Y. Y.; Li Y. Y. Promises of Main Group Metal–Based Nanostructured Materials for Electrochemical CO2 Reduction to Formate. Adv. Energy Mater. 2020, 10 (11), 1902338 10.1002/aenm.201902338.

[ref6] Fan L.; Xia C.; Zhu P.; Lu Y.; Wang H. Electrochemical CO2 Reduction to High-Concentration Pure Formic Acid Solutions in an All-Solid-State Reactor. Nat. Commun. 2020, 11 (1), 1–9. 10.1038/s41467-020-17403-1. 32686669 PMC7371694

[ref7] Yoo J. S.; Christensen R.; Vegge T.; Nørskov J. K.; Studt F. Theoretical Insight into the Trends That Guide the Electrochemical Reduction of Carbon Dioxide to Formic Acid. ChemSusChem 2016, 9 (4), 358–363. 10.1002/cssc.201501197. 26663854

[ref8] Feaster J. T.; Shi C.; Cave E. R.; Hatsukade T.; Abram D. N.; Kuhl K. P.; Hahn C.; Nørskov J. K.; Jaramillo T. F. Understanding Selectivity for the Electrochemical Reduction of Carbon Dioxide to Formic Acid and Carbon Monoxide on Metal Electrodes. ACS Catal. 2017, 7 (7), 4822–4827. 10.1021/acscatal.7b00687.

[ref9] Philips M. F.; Pavesi D.; Wissink T.; Figueiredo M. C.; Gruter G.-J. M.; Koper M. T. M.; Schouten K. J. P. Electrochemical CO2 Reduction on Gas Diffusion Electrodes: Enhanced Selectivity of In–Bi Bimetallic Particles and Catalyst Layer Optimization through a Design of Experiment Approach. ACS Appl. Energy Mater. 2022, 5 (2), 1720–1730. 10.1021/acsaem.1c03156.

[ref10] Shah S. S. A.; Sufyan Javed M.; Najam T.; Molochas C.; Khan N. A.; Nazir M. A.; Xu M.; Tsiakaras P.; Bao S.-J. Metal Oxides for the Electrocatalytic Reduction of Carbon Dioxide: Mechanism of Active Sites, Composites, Interface and Defect Engineering Strategies. Coord. Chem. Rev. 2022, 471, 214716 10.1016/j.ccr.2022.214716.

[ref11] Dutta A.; Kuzume A.; Rahaman M.; Vesztergom S.; Broekmann P. Monitoring the Chemical State of Catalysts for CO2 Electroreduction: An In Operando Study. ACS Catal. 2015, 5 (12), 7498–7502. 10.1021/acscatal.5b02322.

[ref12] Lee C. W.; Cho N. H.; Yang K. D.; Nam K. T. Reaction Mechanisms of the Electrochemical Conversion of Carbon Dioxide to Formic Acid on Tin Oxide Electrodes. ChemElectroChem. 2017, 4 (9), 2130–2136. 10.1002/celc.201700335.

[ref13] Zelocualtecatl Montiel I.; Dutta A.; Kiran K.; Rieder A.; Iarchuk A.; Vesztergom S.; Mirolo M.; Martens I.; Drnec J.; Broekmann P. CO2 Conversion at High Current Densities: Stabilization of Bi(III)-Containing Electrocatalysts under CO2 Gas Flow Conditions. ACS Catal. 2022, 12 (17), 10872–10886. 10.1021/acscatal.2c02549.

[ref14] Luc W.; Collins C.; Wang S.; Xin H.; He K.; Kang Y.; Jiao F. Ag–Sn Bimetallic Catalyst with a Core–Shell Structure for CO2 Reduction. J. Am. Chem. Soc. 2017, 139 (5), 1885–1893. 10.1021/jacs.6b10435. 28094994

[ref15] Cui C.; Han J.; Zhu X.; Liu X.; Wang H.; Mei D.; Ge Q. Promotional Effect of Surface Hydroxyls on Electrochemical Reduction of CO2 over SnOx/Sn Electrode. J. Catal. 2016, 343, 257–265. 10.1016/j.jcat.2015.12.001.

[ref16] Deng W.; Zhang L.; Li L.; Chen S.; Hu C.; Zhao Z.-J.; Wang T.; Gong J. Crucial Role of Surface Hydroxyls on the Activity and Stability in Electrochemical CO2 Reduction. J. Am. Chem. Soc. 2019, 141 (7), 2911–2915. 10.1021/jacs.8b13786. 30715865

[ref17] Li J.; Li J.; Liu X.; Chen J.; Tian P.; Dai S.; Zhu M.; Han Y. F. Probing the Role of Surface Hydroxyls for Bi, Sn and In Catalysts during CO2 Reduction. Appl. Catal. B Environ. 2021, 298 (August), 120581 10.1016/j.apcatb.2021.120581.

[ref18] Lee C. H.; Kanan M. W. Controlling H+ vs CO2 Reduction Selectivity on Pb Electrodes. ACS Catal. 2015, 5 (1), 465–469. 10.1021/cs5017672.

[ref19] Li J.; Jiao J.; Zhang H.; Zhu P.; Ma H.; Chen C.; Xiao H.; Lu Q. Two-Dimensional SnO2 Nanosheets for Efficient Carbon Dioxide Electroreduction to Formate. ACS Sustain. Chem. Eng. 2020, 8 (12), 4975–4982. 10.1021/acssuschemeng.0c01070.

[ref20] Baruch M. F.; Pander J. E.; White J. L.; Bocarsly A. B. Mechanistic Insights into the Reduction of CO2 on Tin Electrodes Using in Situ ATR-IR Spectroscopy. ACS Catal. 2015, 5 (5), 3148–3156. 10.1021/acscatal.5b00402.

[ref21] Detweiler Z. M.; White J. L.; Bernasek S. L.; Bocarsly A. B. Anodized Indium Metal Electrodes for Enhanced Carbon Dioxide Reduction in Aqueous Electrolyte. Langmuir 2014, 30 (25), 7593–7600. 10.1021/la501245p. 24940629

[ref22] Pander J. E.; Baruch M. F.; Bocarsly A. B. Probing the Mechanism of Aqueous CO2 Reduction on Post-Transition-Metal Electrodes Using ATR-IR Spectroelectrochemistry. ACS Catal. 2016, 6 (11), 7824–7833. 10.1021/acscatal.6b01879.

[ref23] Dutta A.; Kuzume A.; Kaliginedi V.; Rahaman M.; Sinev I.; Ahmadi M.; Roldán Cuenya B.; Vesztergom S.; Broekmann P. Probing the Chemical State of Tin Oxide NP Catalysts during CO2 Electroreduction: A Complementary Operando Approach. Nano Energy 2018, 53, 828–840. 10.1016/j.nanoen.2018.09.033.

[ref24] Wang J.; Ning S.; Luo M.; Xiang D.; Chen W.; Kang X.; Jiang Z.; Chen S. In-Sn Alloy Core-Shell Nanoparticles: In-Doped SnOx Shell Enables High Stability and Activity towards Selective Formate Production from Electrochemical Reduction of CO2. Appl. Catal. B Environ. 2021, 288, 119979 10.1016/j.apcatb.2021.119979.

[ref25] Pinheiro Araújo T.; Morales-Vidal J.; Zou T.; García-Muelas R.; Willi P. O.; Engel K. M.; Safonova O. V.; Faust Akl D.; Krumeich F.; Grass R. N.; Mondelli C.; López N.; Pérez-Ramírez J. Flame Spray Pyrolysis as a Synthesis Platform to Assess Metal Promotion in In2O3 -Catalyzed CO2 Hydrogenation. Adv. Energy Mater. 2022, 12 (14), 2103707 10.1002/aenm.202103707.

[ref26] Kortlever R.; Balemans C.; Kwon Y.; Koper M. T. M. Electrochemical CO2 Reduction to Formic Acid on a Pd-Based Formic Acid Oxidation Catalyst. Catal. Today 2015, 244, 58–62. 10.1016/j.cattod.2014.08.001.

[ref27] Gao S.; Lin Y.; Jiao X.; Sun Y.; Luo Q.; Zhang W.; Li D.; Yang J.; Xie Y. Partially Oxidized Atomic Cobalt Layers for Carbon Dioxide Electroreduction to Liquid Fuel. Nature 2016, 529 (7584), 68–71. 10.1038/nature16455. 26738592

[ref28] Solov’eva A. E.; Shvangiradze R. R. Solid Solutions Based on Indium Oxide with Additions of HfO2, CeO2, and TiO2 and Some of Their Properties. Refractories 1995, 36 (7), 219–222. 10.1007/BF02300973.

[ref29] Frei M. S.; Mondelli C.; Cesarini A.; Krumeich F.; Hauert R.; Stewart J. A.; Curulla Ferré D.; Pérez-Ramírez J. Role of Zirconia in Indium Oxide-Catalyzed CO2 Hydrogenation to Methanol. ACS Catal. 2020, 10 (2), 1133–1145. 10.1021/acscatal.9b03305.

[ref30] Wissink T. Electrochemical Reduction of CO2 to Formate on Indium and Bismuth Catalysts; Eindhoven University of Technology, 2023, Vol. 1.

[ref31] Skála T.; Šutara F.; Prince K. C.; Matolín V. Cerium Oxide Stoichiometry Alteration via Sn Deposition: Influence of Temperature. J. Electron Spectrosc. Relat. Phenom. 2009, 169 (1), 20–25. 10.1016/j.elspec.2008.10.003.

[ref32] Kato S.; Ammann M.; Huthwelker T.; Paun C.; Lampimäki M.; Lee M.-T.; Rothensteiner M.; van Bokhoven J. A. Quantitative Depth Profiling of Ce3+ in Pt/CeO2 by in Situ High-Energy XPS in a Hydrogen Atmosphere. Phys. Chem. Chem. Phys. 2015, 17 (7), 5078–5083. 10.1039/C4CP05643D. 25599521

[ref33] Muravev V. Towards Understanding the Catalytic Reactivity of Metal-Ceria Interfaces; Eindhoven University of Technology, 2021, Vol. 1.

[ref34] Ma M.; Clark E. L.; Therkildsen K. T.; Dalsgaard S.; Chorkendorff I.; Seger B. Insights into the Carbon Balance for CO2 Electroreduction on Cu Using Gas Diffusion Electrode Reactor Designs. Energy Environ. Sci. 2020, 13 (3), 977–985. 10.1039/D0EE00047G.

[ref35] Ma M.; Kim S.; Chorkendorff I.; Seger B. Role of Ion-Selective Membranes in the Carbon Balance for CO2 Electroreduction via Gas Diffusion Electrode Reactor Designs. Chem. Sci. 2020, 11 (33), 8854–8861. 10.1039/D0SC03047C. 34123139 PMC8163407

[ref36] Kuhl K. P.; Cave E. R.; Abram D. N.; Jaramillo T. F. New Insights into the Electrochemical Reduction of Carbon Dioxide on Metallic Copper Surfaces. Energy Environ. Sci. 2012, 5 (5), 7050–7059. 10.1039/c2ee21234j.

[ref37] Muravev V.; Spezzati G.; Su Y.-Q.; Parastaev A.; Chiang F.-K.; Longo A.; Escudero C.; Kosinov N.; Hensen E. J. M. Interface Dynamics of Pd–CeO2 Single-Atom Catalysts during CO Oxidation. Nat. Catal. 2021, 4 (6), 469–478. 10.1038/s41929-021-00621-1.

[ref38] WebElements. https://winter.group.shef.ac.uk/webelements (accessed 2024–06–25).

[ref39] Zhu J.; Cannizzaro F.; Liu L.; Zhang H.; Kosinov N.; Filot I. A. W.; Rabeah J.; Brückner A.; Hensen E. J. M. Ni–In Synergy in CO2 Hydrogenation to Methanol. ACS Catal. 2021, 11 (18), 11371–11384. 10.1021/acscatal.1c03170. 34557327 PMC8453486

[ref40] Donley C.; Dunphy D.; Paine D.; Carter C.; Nebesny K.; Lee P.; Alloway D.; Armstrong N. R. Characterization of Indium-Tin Oxide Interfaces Using X-Ray Photoelectron Spectroscopy and Redox Processes of a Chemisorbed Probe Molecule: Effect of Surface Pretreatment Conditions. Langmuir 2002, 18 (2), 450–457. 10.1021/la011101t.

[ref41] Araújo T. P.; Shah A.; Mondelli C.; Stewart J. A.; Curulla Ferré D.; Pérez-Ramírez J. Impact of Hybrid CO2-CO Feeds on Methanol Synthesis over In2O3-Based Catalysts. Appl. Catal. B Environ. 2021, 285, 119878 10.1016/j.apcatb.2021.119878.

[ref42] Pinheiro Araújo T.; Mondelli C.; Agrachev M.; Zou T.; Willi P. O.; Engel K. M.; Grass R. N.; Stark W. J.; Safonova O. V.; Jeschke G.; Mitchell S.; Pérez-Ramírez J. Flame-Made Ternary Pd-In2O3-ZrO2 Catalyst with Enhanced Oxygen Vacancy Generation for CO2 Hydrogenation to Methanol. Nat. Commun. 2022, 13 (1), 1–12. 10.1038/s41467-022-33391-w. 36153333 PMC9509363

[ref43] Hofmann S. Auger- and X-Ray Photoelectron Spectroscopy in Materials Science; Springer Series in Surface Sciences; Springer: Berlin, Heidelberg, 2013; Vol. 49.

[ref44] Pereira-Hernández X. I.; DeLaRiva A.; Muravev V.; Kunwar D.; Xiong H.; Sudduth B.; Engelhard M.; Kovarik L.; Hensen E. J. M.; Wang Y.; Datye A. K. Tuning Pt-CeO2 Interactions by High-Temperature Vapor-Phase Synthesis for Improved Reducibility of Lattice Oxygen. Nat. Commun. 2019, 10 (1), 1358 10.1038/s41467-019-09308-5. 30911011 PMC6433950

[ref45] Mullins D. R. The Surface Chemistry of Cerium Oxide. Surf. Sci. Rep. 2015, 70 (1), 42–85. 10.1016/j.surfrep.2014.12.001.

[ref46] Chen X.; Deng N.; Zhang X.; Li J.; Yang Y.; Hong B.; Jin D.; Peng X.; Wang X.; Ge H.; Jin H. Cerium-Doped Indium Oxide Nanosphere Arrays with Enhanced Ethanol-Sensing Properties. J. Nanoparticle Res. 2019, 21 (4), 77 10.1007/s11051-019-4516-3.

[ref47] Han D.; Song P.; Zhang S.; Zhang H.; Xu Q.; Wang Q. Enhanced Methanol Gas-Sensing Performance of Ce-Doped In2O3 Porous Nanospheres Prepared by Hydrothermal Method. Sensors Actuators, B Chem. 2015, 216, 488–496. 10.1016/j.snb.2015.04.083.

[ref48] Biesinger M. C.; Payne B. P.; Grosvenor A. P.; Lau L. W. M.; Gerson A. R.; Smart R. S. C. Resolving Surface Chemical States in XPS Analysis of First Row Transition Metals, Oxides and Hydroxides: Cr, Mn, Fe, Co and Ni. Appl. Surf. Sci. 2011, 257 (7), 2717–2730. 10.1016/j.apsusc.2010.10.051.

[ref49] Parastaev A.; Muravev V.; Huertas Osta E.; van Hoof A. J. F.; Kimpel T. F.; Kosinov N.; Hensen E. J. M. Boosting CO2 Hydrogenation via Size-Dependent Metal–Support Interactions in Cobalt/Ceria-Based Catalysts. Nat. Catal. 2020, 3 (6), 526–533. 10.1038/s41929-020-0459-4.

[ref50] Davidson A.; Tempere J. F.; Che M.; Roulet H.; Dufour G. Spectroscopic Studies of Nickel(II) and Nickel(III) Species Generated upon Thermal Treatments of Nickel/Ceria-Supported Materials. J. Phys. Chem. 1996, 100 (12), 4919–4929. 10.1021/jp952268w.

[ref51] Biesinger M. C.; Payne B. P.; Lau L. W. M.; Gerson A.; Smart R. S. C. X-Ray Photoelectron Spectroscopic Chemical State Quantification of Mixed Nickel Metal, Oxide and Hydroxide Systems. Surf. Interface Anal. 2009, 41 (4), 324–332. 10.1002/sia.3026.

[ref52] Kibis L. S.; Titkov A. I.; Stadnichenko A. I.; Koscheev S. V.; Boronin A. I. X-Ray Photoelectron Spectroscopy Study of Pd Oxidation by RF Discharge in Oxygen. Appl. Surf. Sci. 2009, 255 (22), 9248–9254. 10.1016/j.apsusc.2009.07.011.

[ref53] Muravev V.; Parastaev A.; van den Bosch Y.; Ligt B.; Claes N.; Bals S.; Kosinov N.; Hensen E. J. M. Size of Cerium Dioxide Support Nanocrystals Dictates Reactivity of Highly Dispersed Palladium Catalysts. Science (80-.). 2023, 380 (6650), 1174–1179. 10.1126/science.adf9082. 37319196

[ref54] Toyoshima R.; Yoshida M.; Monya Y.; Kousa Y.; Suzuki K.; Abe H.; Mun B. S.; Mase K.; Amemiya K.; Kondoh H. In Situ Ambient Pressure XPS Study of CO Oxidation Reaction on Pd(111) Surfaces. J. Phys. Chem. C 2012, 116 (35), 18691–18697. 10.1021/jp301636u.

[ref55] Wissink T.; van de Poll R. C. J.; Figueiredo M. C.; Hensen E. J. M. Stability of In2O3 Nanoparticles in PTFE-Containing Gas Diffusion Electrodes for CO2 Electroreduction to Formate. J. CO2 Util. 2023, 67, 102331 10.1016/j.jcou.2022.102331.

[ref56] Diercks J. S.; Herranz J.; Georgi M.; Diklić N.; Chauhan P.; Ebner K.; Clark A. H.; Nachtegaal M.; Eychmüller A.; Schmidt T. J. Interplay between Surface-Adsorbed CO and Bulk Pd Hydride under CO2-Electroreduction Conditions. ACS Catal. 2022, 12 (17), 10727–10741. 10.1021/acscatal.2c02660.

[ref57] Wang T.-J.; Fang W.-S.; Liu Y.-M.; Li F.-M.; Chen P.; Chen Y. Heterostructured Pd/PdO Nanowires for Selective and Efficient CO2 Electroreduction to CO. J. Energy Chem. 2022, 70, 407–413. 10.1016/j.jechem.2022.03.001.

[ref58] Grujicic D.; Pesic B. Electrochemical and AFM Study of Cobalt Nucleation Mechanisms on Glassy Carbon from Ammonium Sulfate Solutions. Electrochim. Acta 2004, 49 (26), 4719–4732. 10.1016/j.electacta.2004.05.028.

[ref59] Shulga Y. M.; Vasilets V. N.; Kiryukhin D. P.; Voylov D. N.; Sokolov A. P. Polymer Composites Prepared by Low-Temperature Post-Irradiation Polymerization of C2F4 in the Presence of Graphene-like Material: Synthesis and Characterization. RSC Adv. 2015, 5 (13), 9865–9874. 10.1039/C4RA09074H.

[ref60] Peng X.; Jin X.; Gao B.; Liu Z.; Chu P. K. Strategies to Improve Cobalt-Based Electrocatalysts for Electrochemical Water Splitting. J. Catal. 2021, 398, 54–66. 10.1016/j.jcat.2021.04.003.

[ref61] Huang C.; Qin P.; Luo Y.; Ruan Q.; Liu L.; Wu Y.; Li Q.; Xu Y.; Liu R.; Chu P. K. Recent Progress and Perspective of Cobalt-Based Catalysts for Water Splitting: Design and Nanoarchitectonics. Mater. Today Energy 2022, 23, 100911 10.1016/j.mtener.2021.100911.

[ref62] Feng X.; Sayle D. C.; Wang Z. L.; Paras M. S.; Santora B.; Sutorik A. C.; Sayle T. X. T.; Yang Y.; Ding Y.; Wang X.; Her Y.-S. Converting Ceria Polyhedral Nanoparticles into Single-Crystal Nanospheres. Science (80-.). 2006, 312 (5779), 1504–1508. 10.1126/science.1125767. 16763144

[ref63] Stetsovych V.; Pagliuca F.; Dvořák F.; Duchoň T.; Vorokhta M.; Aulická M.; Lachnitt J.; Schernich S.; Matolínová I.; Veltruská K.; Skála T.; Mazur D.; Mysliveček J.; Libuda J.; Matolín V. Epitaxial Cubic Ce2O3 Films via Ce–CeO2 Interfacial Reaction. J. Phys. Chem. Lett. 2013, 4 (6), 866–871. 10.1021/jz400187j. 26291348

[ref64] Hirosaki N.; Ogata S.; Kocer C. Ab Initio Calculation of the Crystal Structure of the Lanthanide Ln2O3 Sesquioxides. J. Alloys Compd. 2003, 351 (1–2), 31–34. 10.1016/S0925-8388(02)01043-5.

[ref65] Marezio M. Refinement of the Crystal Structure of In2O3 at Two Wavelengths. Acta Crystallogr. 1966, 20 (6), 723–728. 10.1107/S0365110X66001749.

[ref66] Magari Y.; Yeh W.; Ina T.; Furuta M. Influence of Grain Boundary Scattering on the Field-Effect Mobility of Solid-Phase Crystallized Hydrogenated Polycrystalline In2O3 (In2O3:H). Nanomaterials 2022, 12 (17), 2958 10.3390/nano12172958. 36079995 PMC9458122

[ref67] Maeno Z.; Yasumura S.; Liu C.; Toyao T.; Kon K.; Nakayama A.; Hasegawa J.; Shimizu K. Experimental and Theoretical Study of Multinuclear Indium–Oxo Clusters in CHA Zeolite for CH4 Activation at Room Temperature. Phys. Chem. Chem. Phys. 2019, 21 (25), 13415–13427. 10.1039/C9CP01873E. 31093622

[ref68] Liang Z.; Song L.; Sun M.; Huang B.; Du Y. Atomically Dispersed Indium and Cerium Sites for Selectively Electroreduction of CO2 to Formate. Nano Res. 2023, 16, 8757 10.1007/s12274-023-5481-9.

[ref100] Piro N. A.; Robinson J. R.; Walsh P. J.; Schelter E. J. The Electrochemical Behavior of Cerium(III/IV) Complexes: Thermodynamics, Kinetics and Applications in Synthesis. Coord. Chem. Rev. 2014, 260 (1), 21–36. 10.1016/j.ccr.2013.08.034.

[ref101] Saravanan T.; Shanmugam M.; Anandan P.; Azhagurajan M.; Pazhanivel K.; Arivanandhan M.; Hayakawa Y.; Jayavel R. Facile Synthesis of Graphene-CeO_2_ Nanocomposites with Enhanced Electrochemical Properties for Supercapacitors. Dalton Trans. 2015, 44 (21), 9901–9908. 10.1039/C5DT01235J. 25940081

[ref69] Chi L.-P.; Niu Z.-Z.; Zhang X.-L.; Yang P.-P.; Liao J.; Gao F.-Y.; Wu Z.-Z.; Tang K.-B.; Gao M.-R. Stabilizing Indium Sulfide for CO2 Electroreduction to Formate at High Rate by Zinc Incorporation. Nat. Commun. 2021, 12 (1), 5835 10.1038/s41467-021-26124-y. 34611149 PMC8492718

