# Supplementary material for: Ce Promotion of In2O3 for Electrochemical Reduction of CO2 to Formate
Source: ACS Catal. 2024 Oct 25;14(22):16589–604. doi: 10.1021/acscatal.4c02619 (PMC11575495; doi:10.1021/acscatal.4c02619)
Supplement: Supplementary file 1 — cs4c02619_si_001.docx [file cs4c02619_si_001.docx]

Supporting information for:

Ce promotion of In_2_O_3_ for electrochemical reduction of CO_2_ to formate

Tim Wissink^a^, Floriane A. Rollier^a^, Valery Muravev^a^, Jason M.J.J. Heinrichs^a^, Rim C.J. van de Poll^a^, Jiadong Zhu^a^, Dimitra Anastasiadou^a^, Nikolay Kosinov^a^, Marta C. Figueiredo^a^, Emiel J.M. Hensen^a*^

^a^ Laboratory of Inorganic Materials and Catalysis, Department of Chemical Engineering and Chemistry, Eindhoven University of Technology, P.O. Box 513, 5600 MB Eindhoven, The Netherlands

* Corresponding author.

*E-mail address:* [E.J.M.Hensen@tue.nl](mailto:E.J.M.Hensen@tue.nl) (E.J.M. Hensen)

# Catalyst Synthesis and Characterization

*
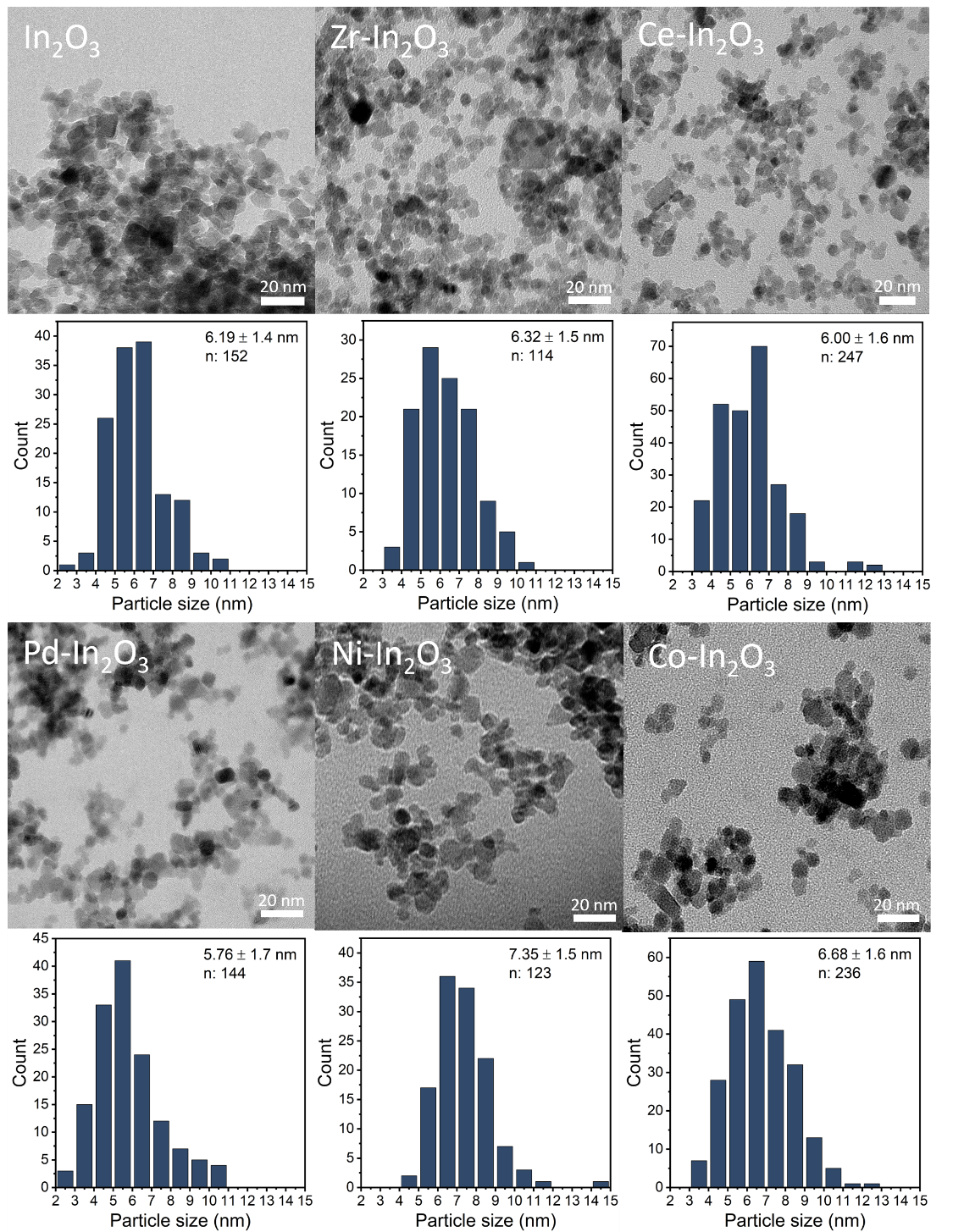
*

Figure S1: TEM images and corresponding size distributions of the FSP synthesized doped In_2_O_3_ nanoparticles. The size distribution of the nanoparticles is obtained counting no less than 100 particles per sample. The TEM analysis of pure In_2_O_3_ was adapted from previous literature.^1^





Figure S2: XRD diffractograms of the doped In2O3 nanoparticles, the diffraction lines are characterized according to PDF: 00-044-1087, belonging to the cubic In2O3 phase. The X-ray diffractogram of pure In_2_O_3_ was adapted from previous literature.^1^


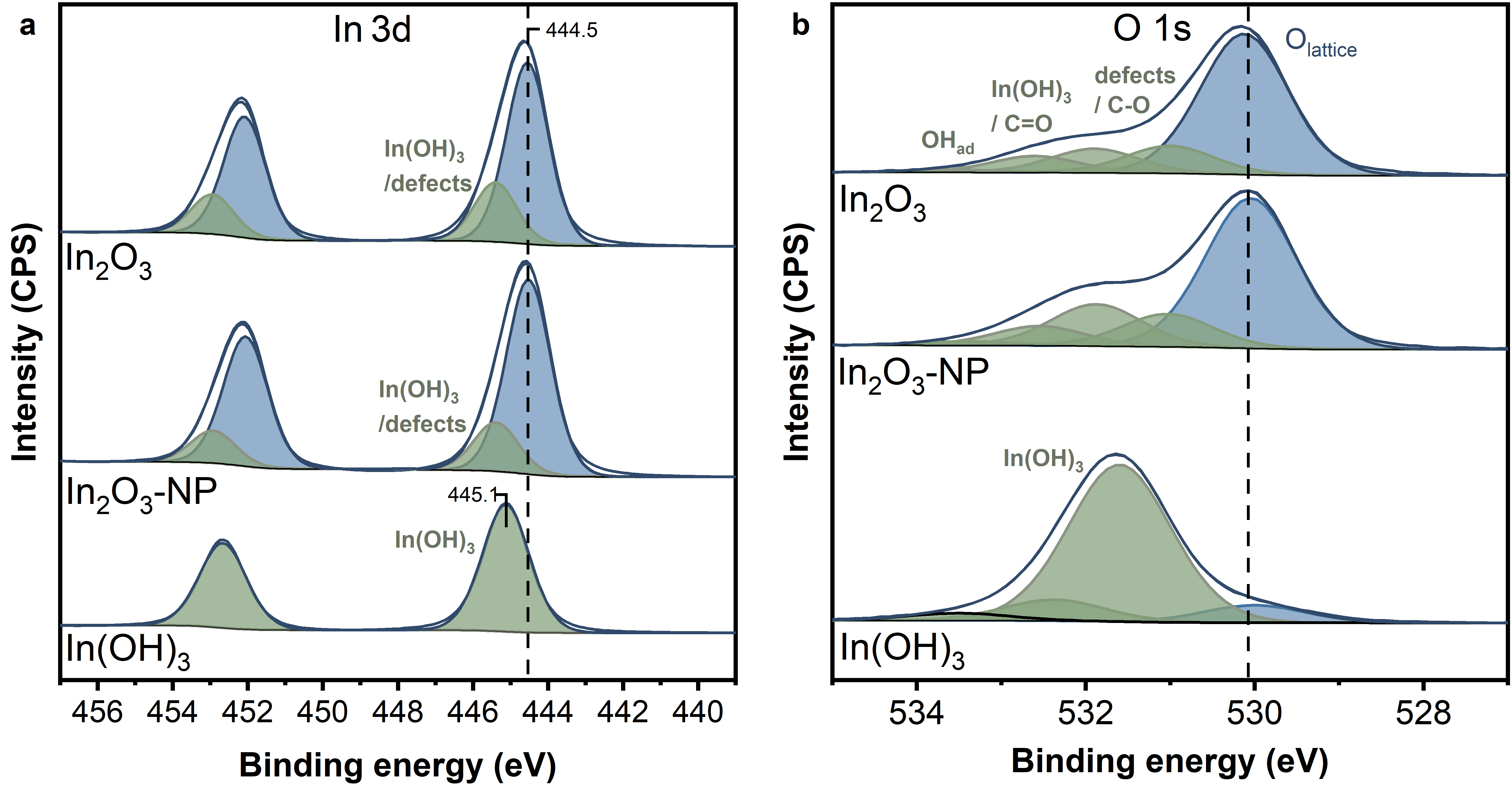


Figure S3: XPS spectra of the In 3d core-line region (a) and O 1s core-line region (b) of reference In_2_O_3_ (Sigma Aldrich), In(OH)_3_ (Alfa Aesar), and the FSP synthesized In_2_O_3_ nanoparticles.


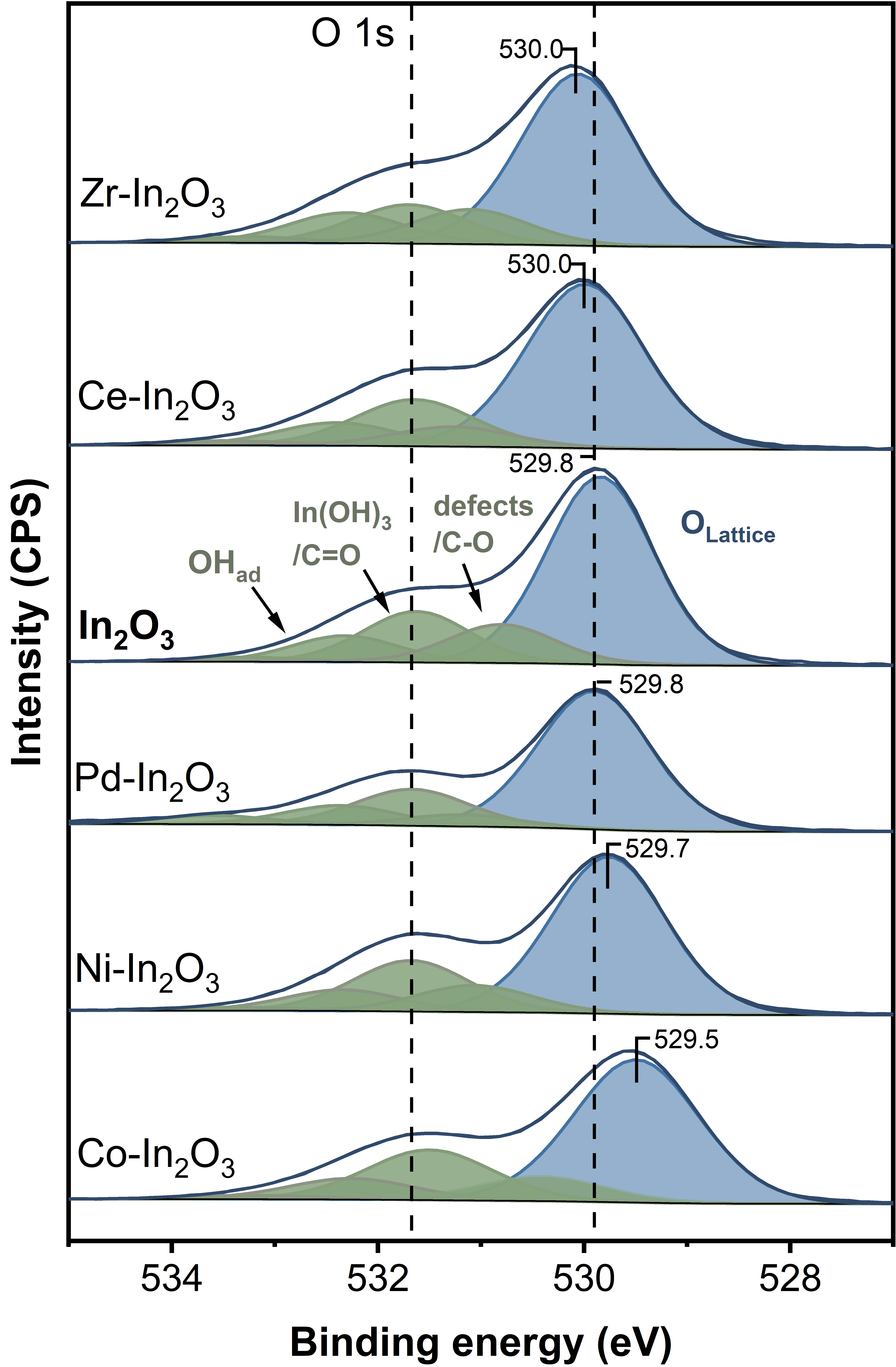


Figure S4: XPS spectra of the O 1s core-line region of the FSP synthesized (doped) In_2_O_3_. All spectra were energy corrected by the U’’’ component at 916.7 eV of Ce.^2^ The resulting C1s binding energy (measured at 285.1 eV) was used to energy correct spectra of samples that contain no Ce.

*
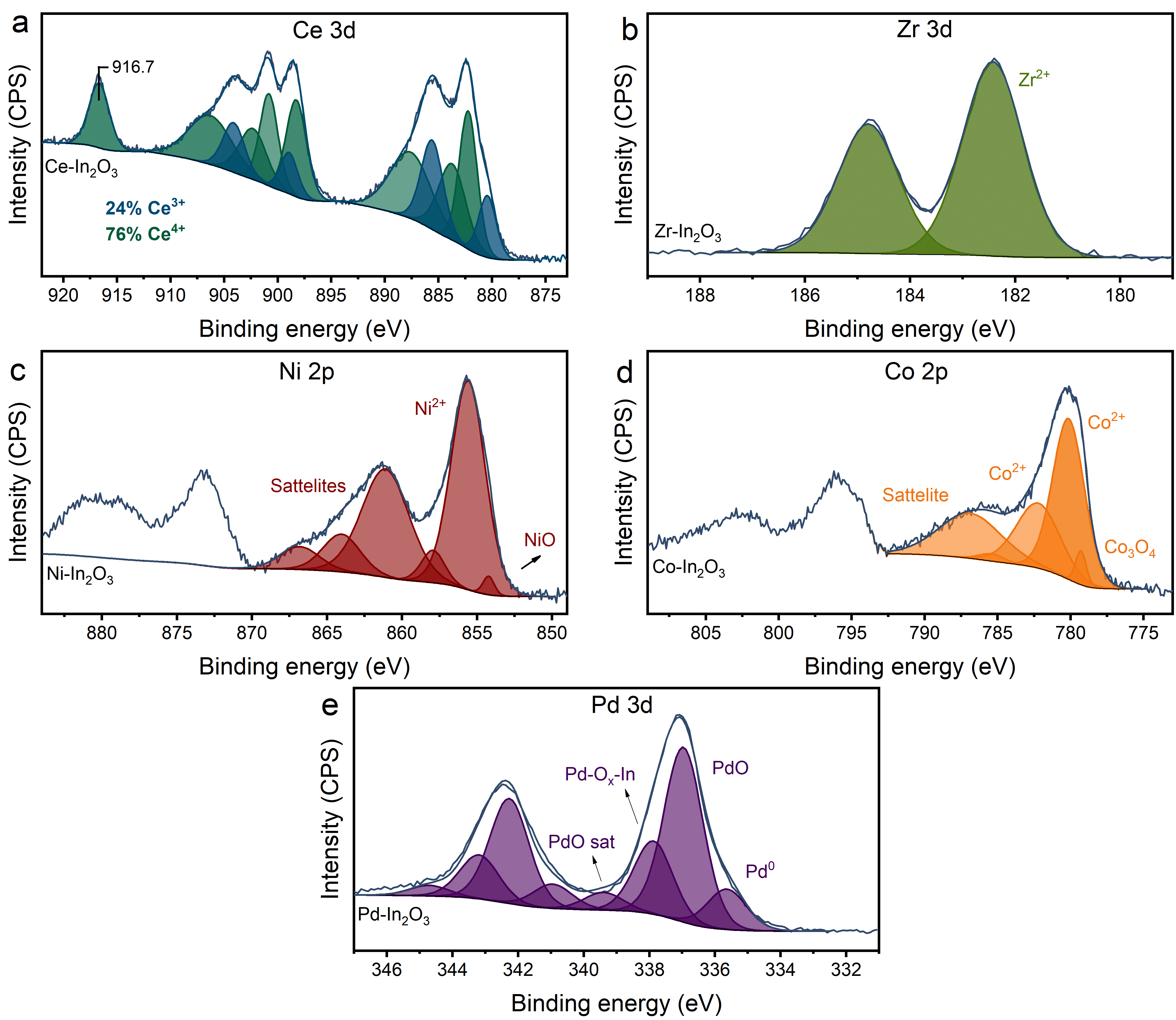
*

Figure S5: XPS spectra of the Ce 3d (a), Zr 3d (b), Ni 2p (c), Co 2p (d) and Pd 3d (e) core-line regions of the as prepared FSP-synthesized doped In_2_O_3_ nanoparticles. All spectra were energy corrected by the U’’’ component at 916.7 eV of Ce.^2^ The resulting C1s binding energy (measured at 285.1 eV) was used to energy correct spectra of samples that contain no Ce. Pd deconvolution was performed including a metallic Pd, PdO and oxygen deficient PdO contribution.^3,4,5^ The Co 2p region was fitted with Co^2+^ contributions originating from CoO or Co(OH)_2_ and a minor contribution of fully oxidized Co_3_O_4_.^6^ The Ni 2p region was fitted by with Ni^2+^ contributions, most likely originating from surface Ni(OH)_2_ or NiO clusters with a minor contribution of NiO.^7,8,9^

# Electrocatalytic CO_2_ reduction


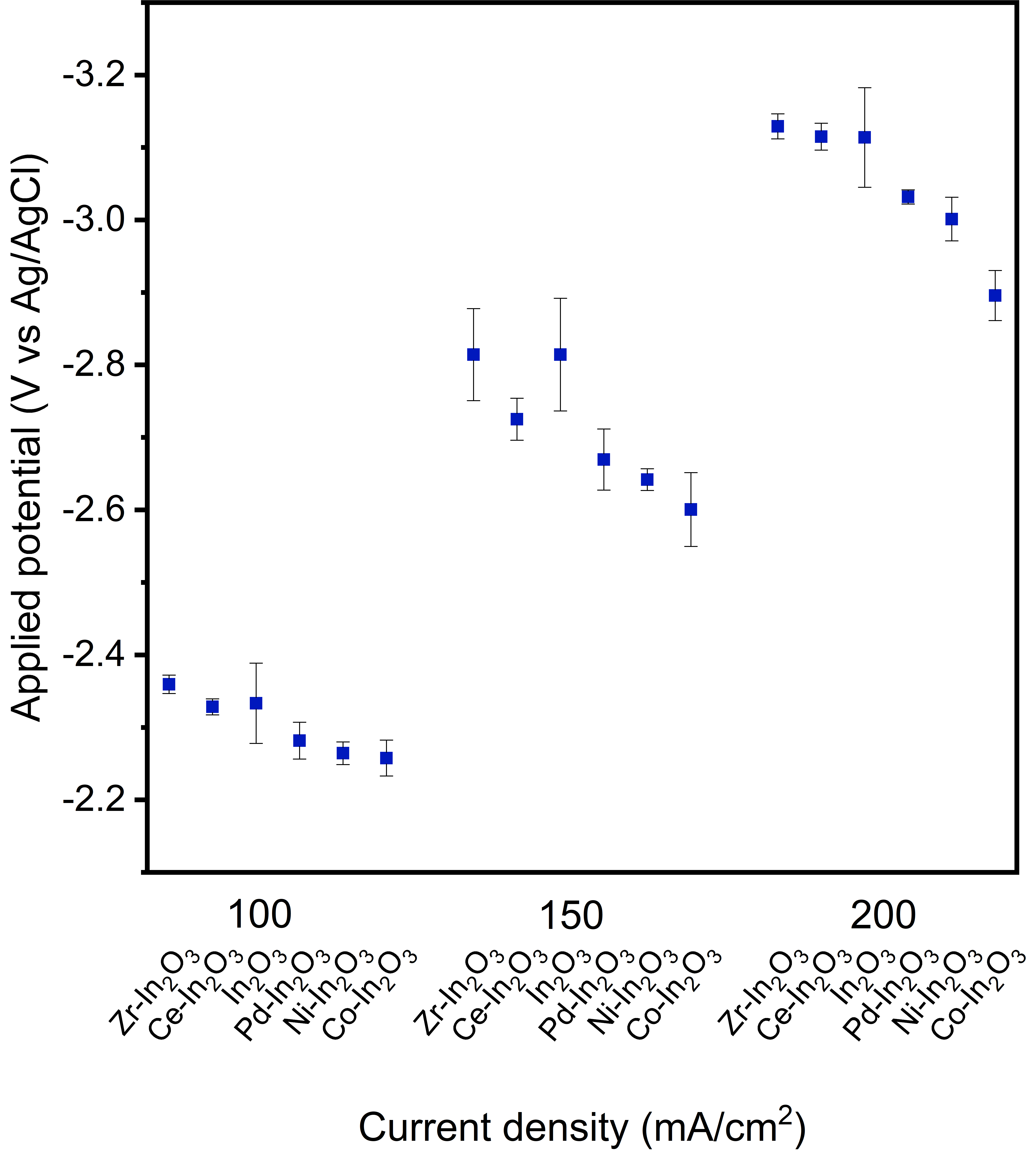


Figure S6: Average applied potential as a function of imposed current density on doped In_2_O_3_ catalysts during 1 h CO_2_ER. Experiments were repeated in triplicate to obtain the error bars. A decrease in current density is observed with increasing FE towards hydrogen, most likely due to decrease in kinetic barrier for hydrogen evolution on the increasingly metallic character of the catalyst or large surface content of dopants on the In_2_O_3_ nanoparticles.


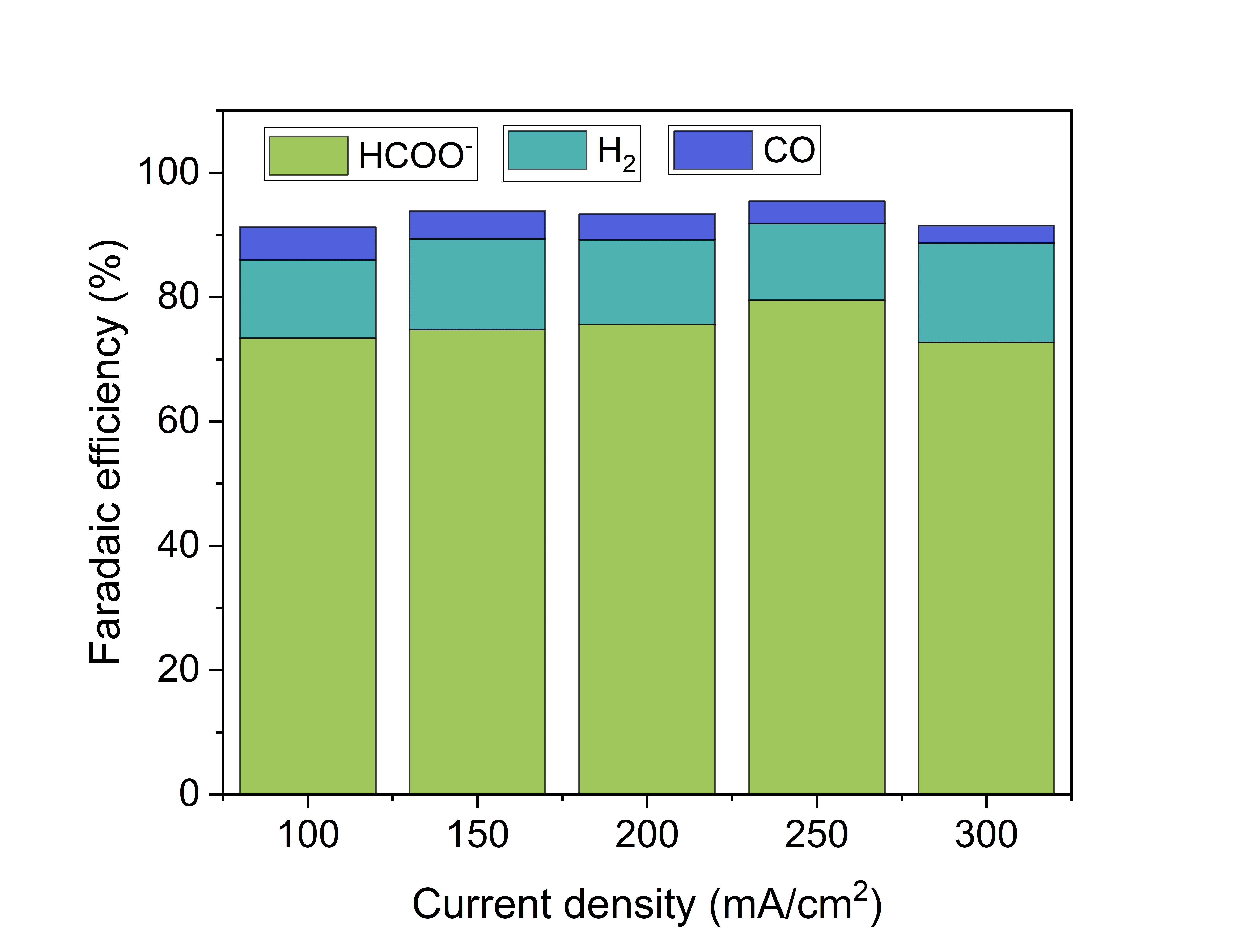


Figure S7: Faradaic efficiency towards formate, H_2_ and CO as a function of imposed current density on In_2_O_3_ after 1 h CO_2_ER. Catalyst loading: 0.5 mg/cm^2^.

Table S1: XPS data quantification from GDE’s, surface dopant content is calculated as percentage from the fraction of dopant atoms / (dopant + In atoms)

| **In_2_O_3_** | **-** | **In^0^ (At.%)** | **In_2_O_3_ (At.%)** | **In(OH)_3_ (At.%)** |
| --- | --- | --- | --- | --- |
| **As prepared** | - | 0 | 88.7 | 11.3 |
| **1 h 100 mA** | - | 10.6 | 81.4 | 8.0 |
|  | | | | |
| **Ce-In_2_O_3_** | **Ce (At.%)** | **In^0^ (At.%)** | **In_2_O_3_ (At.%)** | **In(OH)_3_ (At.%)** |
| **As prepared** | 10.1 | 0 | 89.9 | 10.1 |
| **1 h 100 mA** | 5.6 | 7.2 | 83.4 | 9.4 |
|  | | | | |
| **Co-In_2_O_3_** | **Co (At.%)** | **Co-In alloy (At.%)** | **In_2_O_3_ (At.%)** | **In(OH)_3_ (At.%)** |
| **As prepared** | 12.4 | 0 | 75.8 | 24.2 |
| **1 h 100 mA** | 14.5 | 73.22 | 0 | 26.78 |


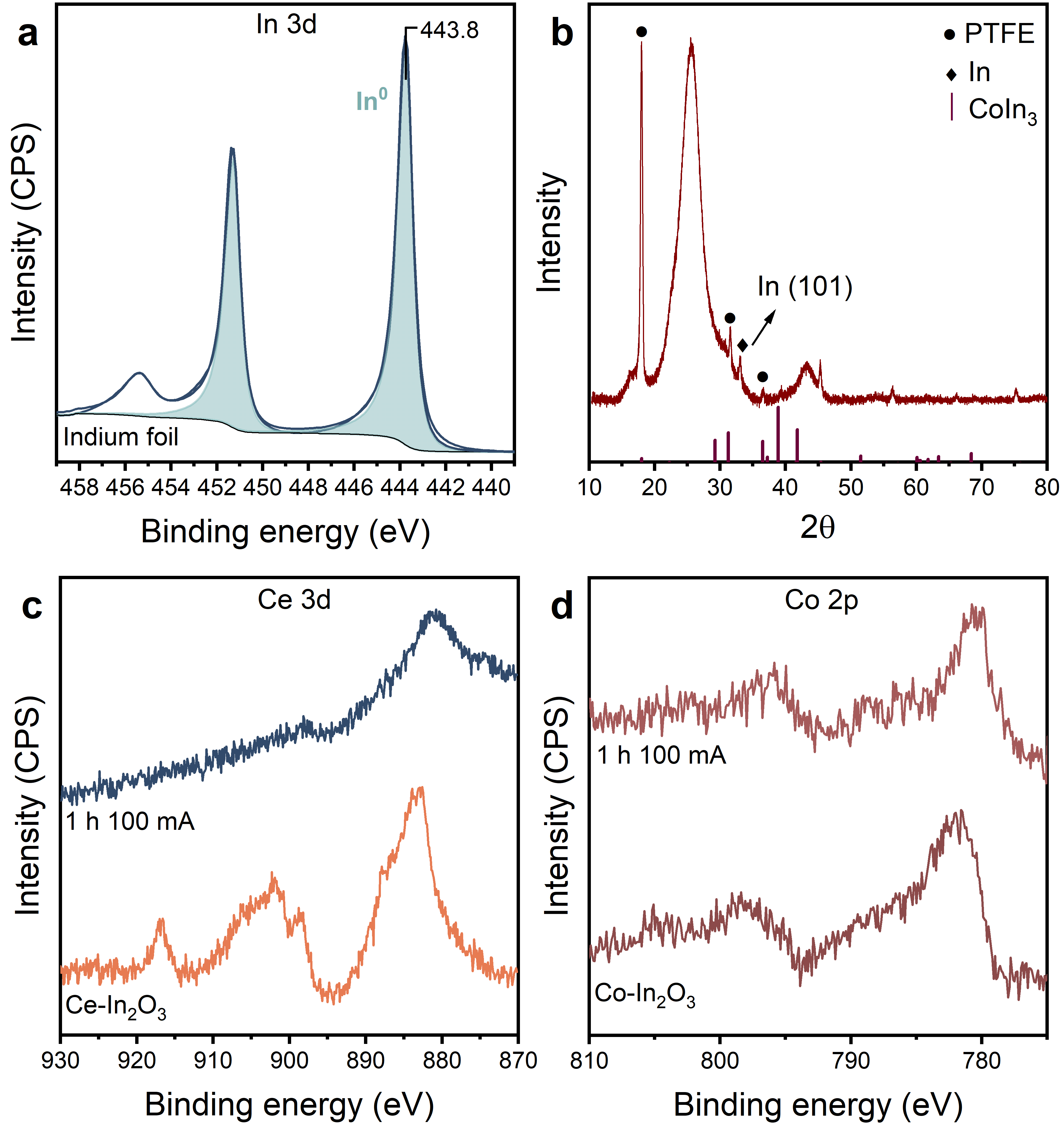


Figure S8: (a) XPS analysis of the In 3d core-line region of etched In foil. (b) XPS analysis of the Ce 3d core-line region before (orange) and after (blue) CO_2_ER using a GDE with Ce-In_2_O_3_ catalyst. (c) The Co 2p core-line region before (brown) and after (red) CO_2_ER using a GDE with Ce-In_2_O_3_ catalyst. (d) XRD diffractogram on the GDE after 1 h CO_2_ER, except for metallic In, no clear peaks belonging to In_2_O_3_ or an In-Co alloy could be observed, possibly due to the relatively high PTFE and carbon content with respect to catalyst.

# Ce-In2O3 catalysts

*
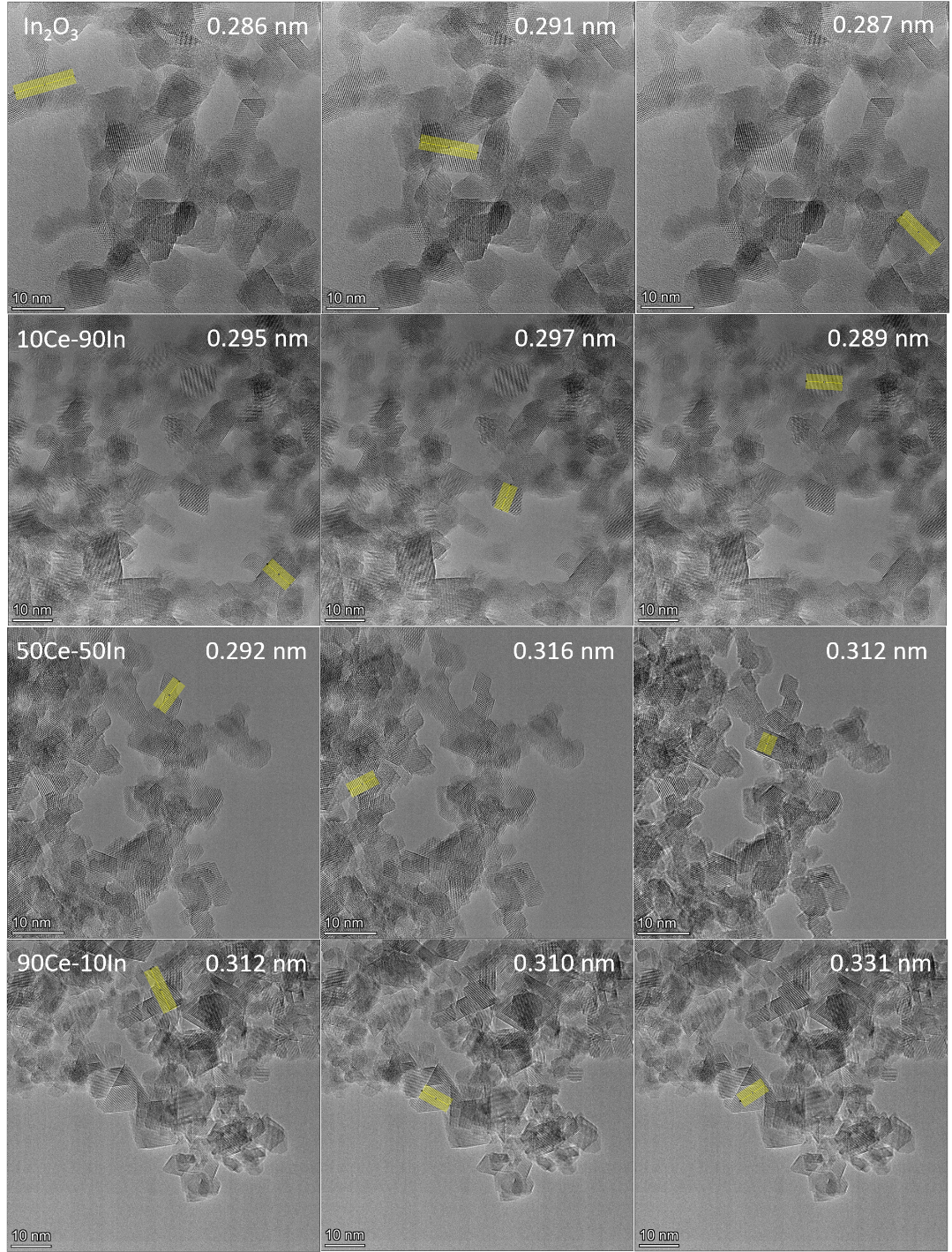
*

Figure S9: HRTEM images of the FSP synthesized In_2_O_3_ and Ce-In catalysts. Lattice parameters are determined from averaging at least 20 lines. In general, an increasing lattice constant is observed with increasing Ce content in the nanoparticles.


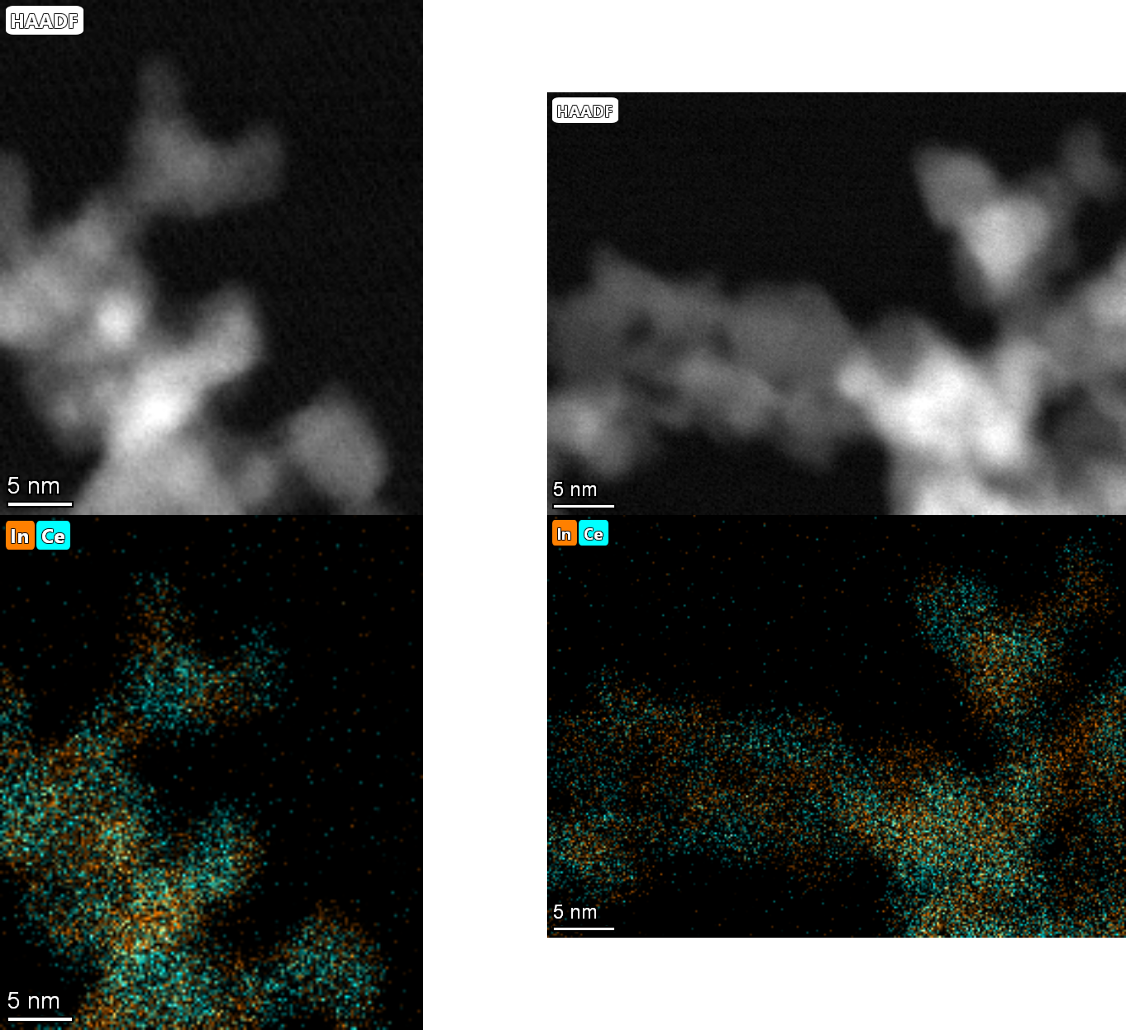


Figure S10: (a) STEM-EDX images on two different agglomerations of particles of the as prepared Ce(50)-In_2_O_3_ sample (Top: High-angle annular dark-field (HAADF) images, bottom: EDX mapping). A high dispersion of Ce and In atoms and clusters in the particles is observed. No apparent core shell structure with an In enriched surface can be distinguished.

*
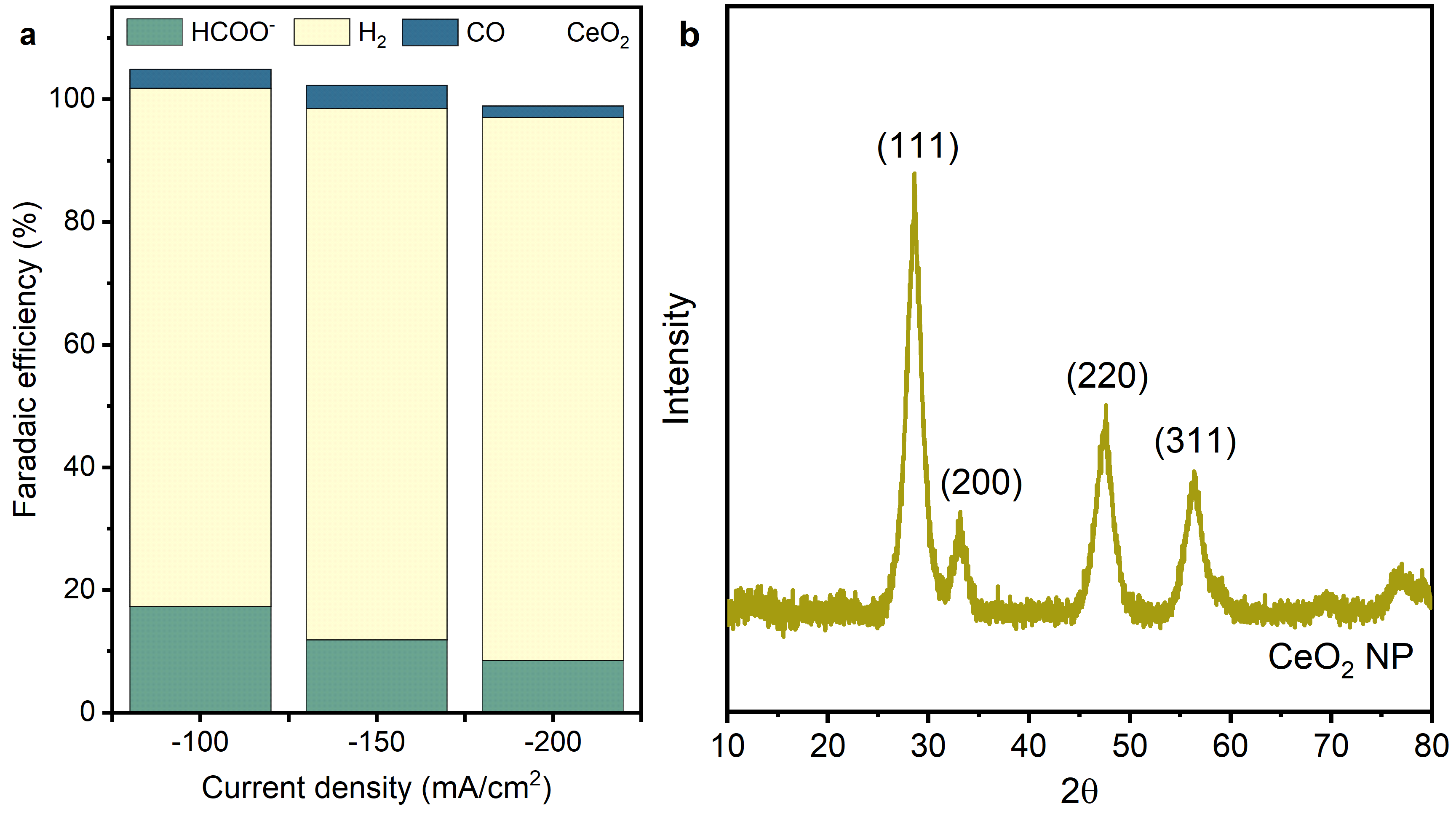
*

Figure S11: (a) Faradaic efficiency as a function of current density on CeO_2_ after 1 h CO_2_ER. (b) XRD diffractogram of FSP-synthesized CeO_2_ nanoparticles (~4 nm).

*
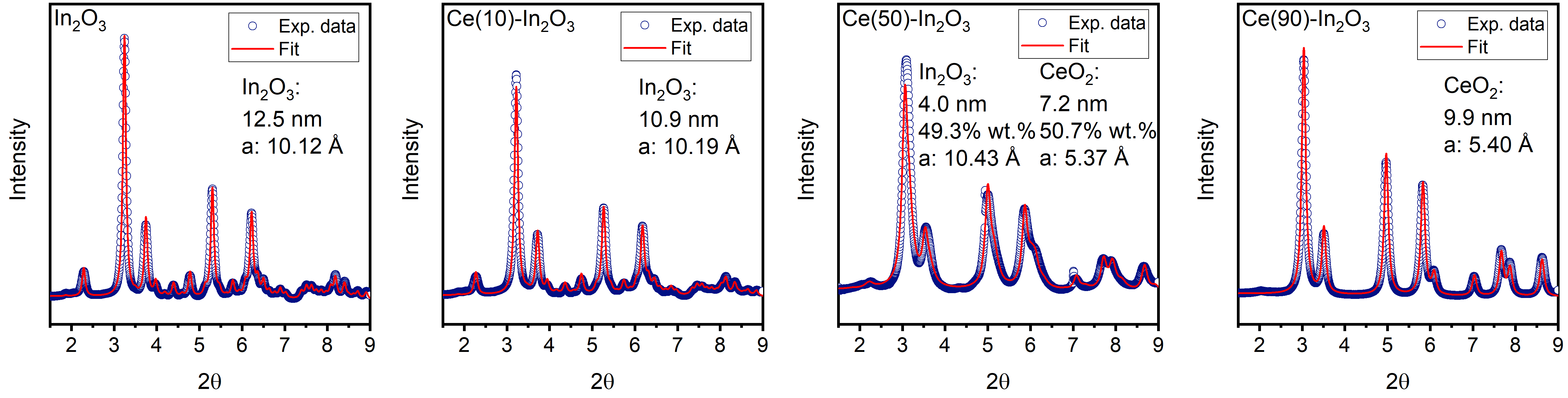
*

Figure S12: Rietveld refinement on synchrotron-based XRD (sXRD) patterns of the powders of as-prepared catalysts. The change in lattice parameter ‘a’ of the unit cell is determined for the fitted In_2_O_3_ (a = 10.117 Å) and CeO_2_ (a = 5.412 Å) phases. Rietveld refinement appears to overestimate the particle size, the error could be due to the high correlation between lattice strain and particle size.


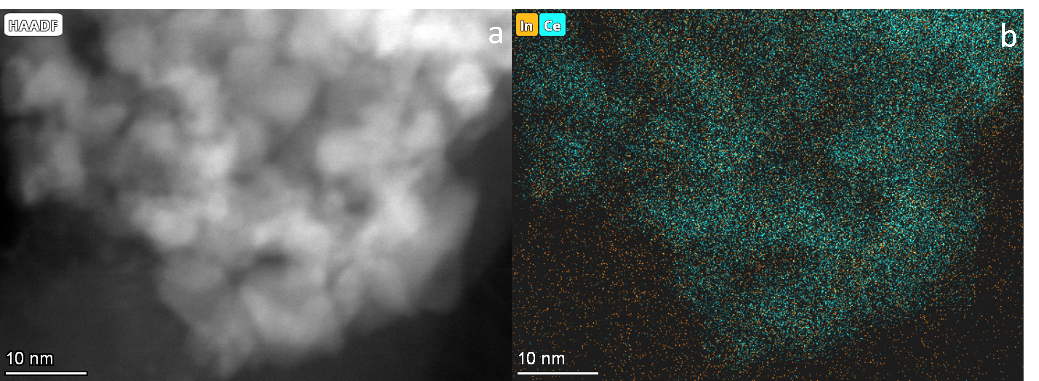


Figure S13: STEM-EDX image of the used Ce(90)-In_2_O_3_ electrode. (a) High-angle annular dark-field (HAADF) image, (b) EDX mapping. The In atoms are highly dispersed over the CeO_2_ particles. Additionally, In atoms are observed on the carbon support around the CeO_2_ particles, originating from dissolution and redeposition of In.


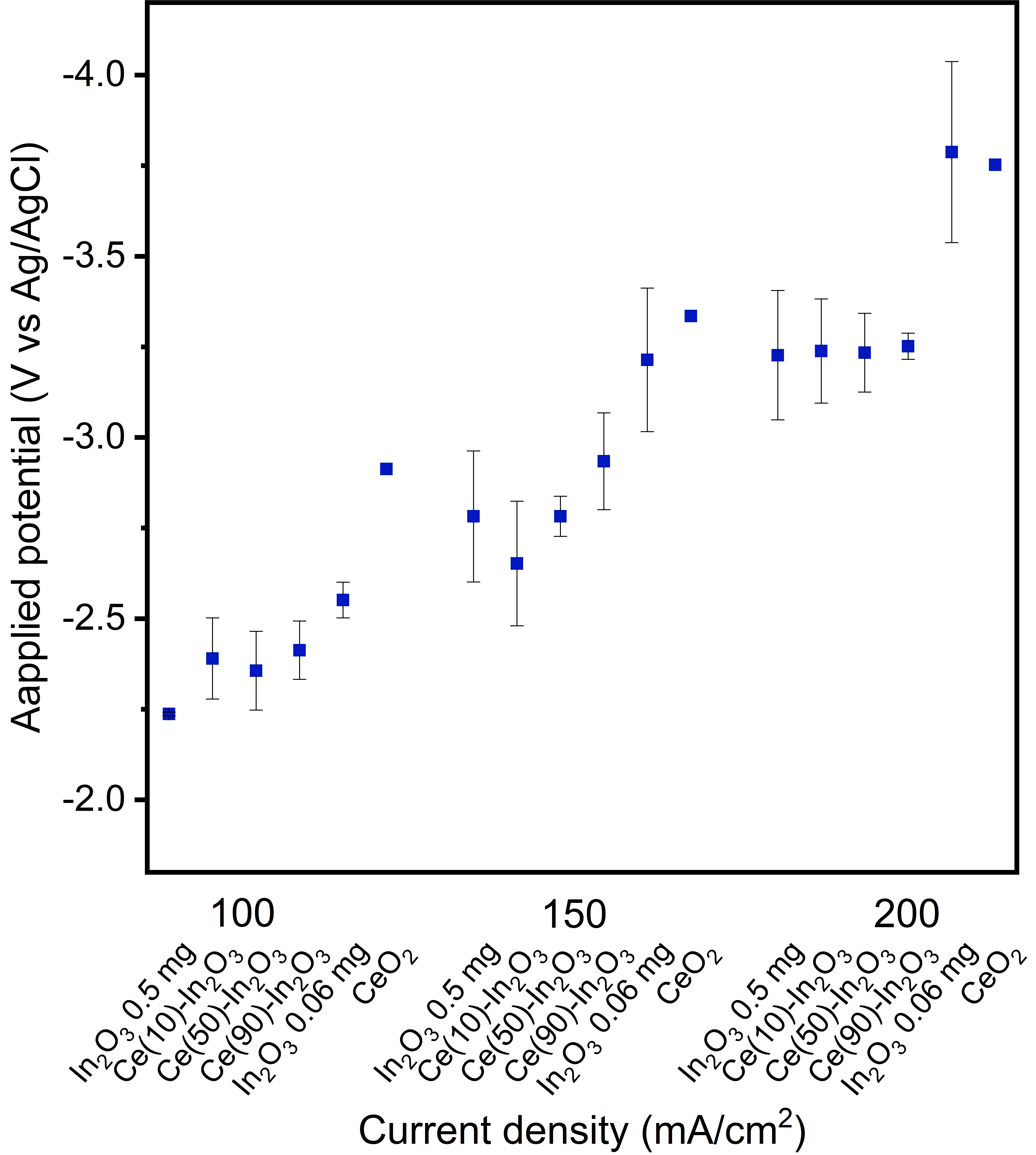


Figure S14: Average applied potential as a function of imposed current density on Ce-In_2_O_3_ catalysts during 1 h CO_2_ER. Experiments were repeated in duplicate to obtain the standard deviations.

*
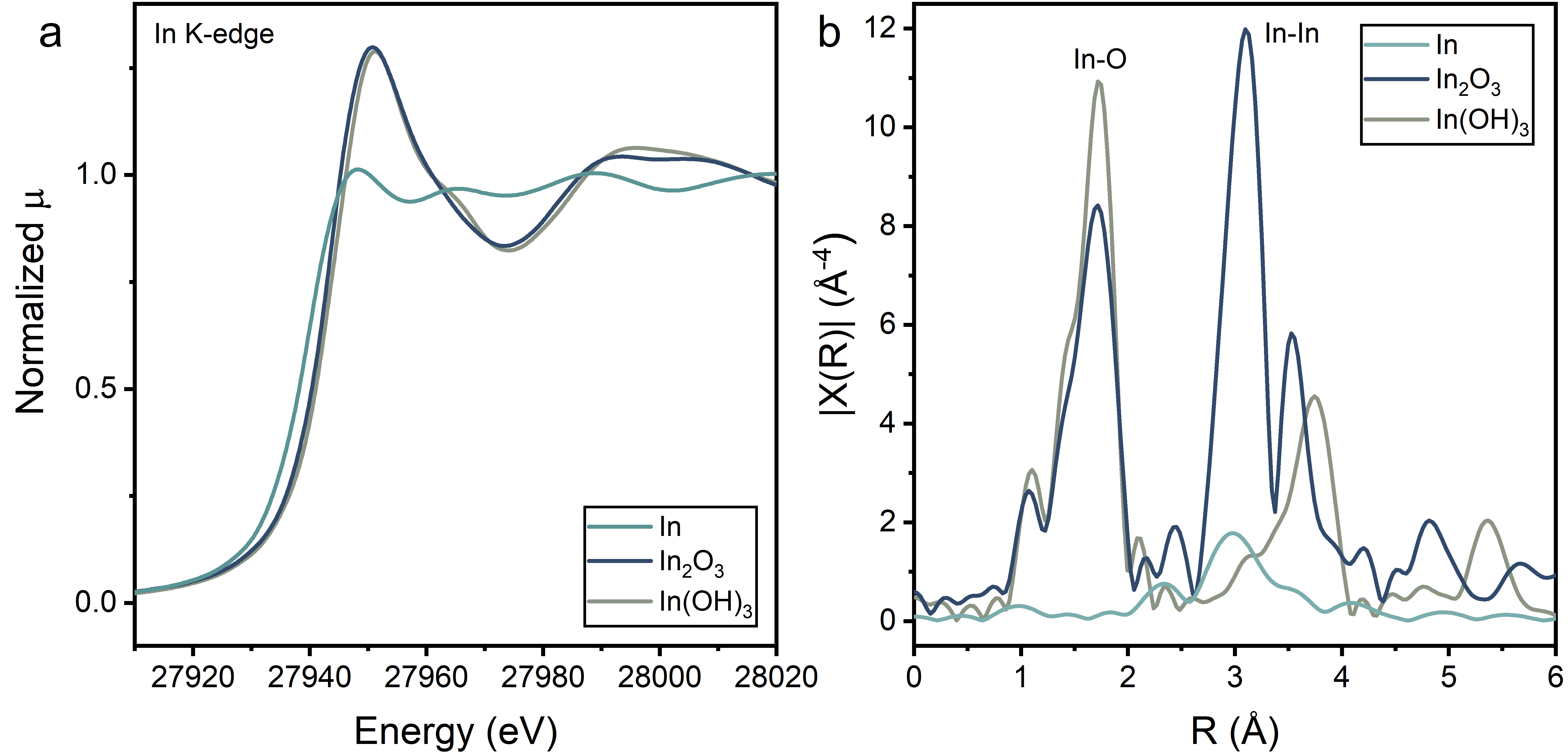
*

Figure S15: XANES (a) and k^3^ weighted FT-EXAFS (b) spectral region of the In K-edge of In foil, In_2_O_3_ nanoparticles, and reference In(OH)_3_.

*
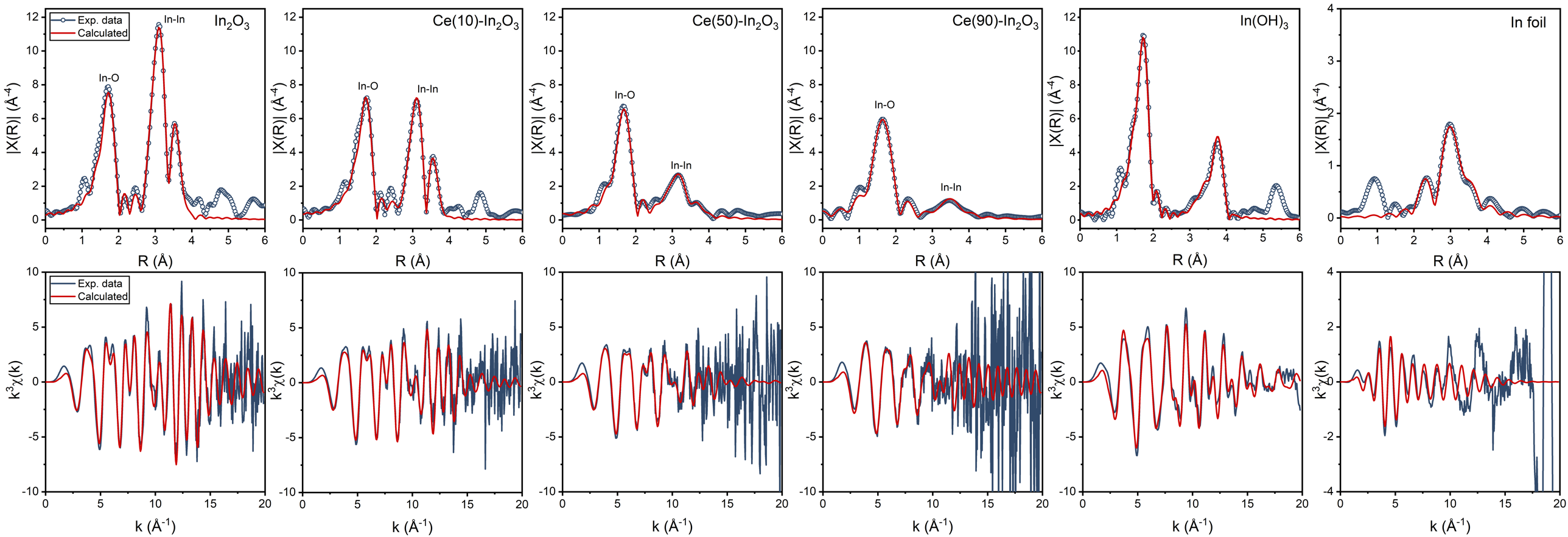
*

Figure S16: The k^3^ weighted FT-EXAFS spectra (In K-edge) of pure In_2_O_3_ and Ce-In_2_O_3_ nanoparticles before CO_2_ER and In-foil and In(OH)_3_ references. The S_0_^2^ value (0.77) was determined by fitting the two In-In scattering paths of the first shell of reference In foil, fixing the coordination numbers to respectively 4 and 8. ΔE_0_ and ΔR values were shared for all the shells and ΔR was limited to max. 0.08 for Ce(50)-In_2_O_3_ and Ce(90)-In_2_O_3_. The coordination number of the second In-In shell decreased from 11.8 ± 1.5 in In_2_O_3_ to 4.1 ± 3.0 in Ce(90)-In_2_O_3_. The errors indicate the uncertainties of fitting results derived from Larch software.

Table S2: EXAFS fitting results

| **Sample** | **Path** | **R [Å]** | **CN** | **σ^2^ [Å^2^]** | **ΔE_0_ [eV]** | **R-factor (%)** |
| --- | --- | --- | --- | --- | --- | --- |
| **In foil** | In-In | 3.18 ± 0.02 | 4.0 | 0.012 ± 0.003 | 5.50 ± 1.16 | 8.0 |
|  | In-In | 3.35 ± 0.03 | 8.0 | 0.020 ± 0.001 |  |  |
| **In_2_O_3_** | In-O | 2.16 ± 0.01 | 6.0 ± 0.8 | 0.005 ± 0.001 | 7.11 ± 0.74 | 1.3 |
|  | In-In (oxide) | 3.36 ± 0.01 | 5.9 ± 0.6 | 0.004 ± 0.001 |  |  |
|  | In-In (oxide) | 3.85 ± 0.01 | 5.9 ± 1.5 | 0.007 ± 0.001 |  |  |
| **Ce(10)-In_2_O_3_** | In-O | 2.16 ± 0.01 | 6.0 ± 0.6 | 0.005 ± 0.001 | 5.90 ± 0.83 | 2.0 |
|  | In-In (oxide) | 3.37 ± 0.08 | 5.8 ± 0.8 | 0.006 ± 0.001 |  |  |
|  | In-In (oxide) | 3.85 ± 0.07 | 3.8 ± 1.4 | 0.007 ± 0.002 |  |  |
| **Ce(50)-In_2_O_3_** | In-O | 2.15 ± 0.01 | 5.9 ± 5.2 | 0.006 ± 0.001 | 6.70 ± 1.17 | 1.5 |
|  | In-In (oxide) | 3.37 ± 0.01 | 4.4 ± 1.4 | 0.008 ± 0.002 |  |  |
|  | In-In (oxide) | 3.84 ± 0.02 | 3.1 ± 2.9 | 0.010 ± 0.006 |  |  |
| **Ce(90)-In_2_O_3_** | In-O | 2.15 ± 0.01 | 6.0 ± 2.1 | 0.006 ± 0.002 | 7.11 ± 1.61 | 1.4 |
|  | In-In (oxide) | 3.37 ± 0.03 | 2.7 ± 3.0 | 0.010 ± 0.012 |  |  |
|  | In-In (oxide) | 3.85 ± 0.03 | 1.4 ± 2.9 | 0.002 ± 0.016 |  |  |
| **In_2_O_3_ @ -2.0 V** | In-O | 2.02 ± 0.05 | 0.8 ± 0.5 | 0.004 ± 0.008 | -11.8 ± 9.6 | 0.6 |
| **In(OH)_3_** | In-O | 2.16 ± 0.01 | 6.0 | 0.004 ± 0.001 | 8.14 ± 0.74 | 5.0 |
|  | In-O (oxide) | 3.94 ± 0.04 | 6.0 | 0.006 ± 0.005 |  |  |
|  | In-In | 4.00 ± 0.01 | 6.0 | 0.006 ± 0.001 |  |  |


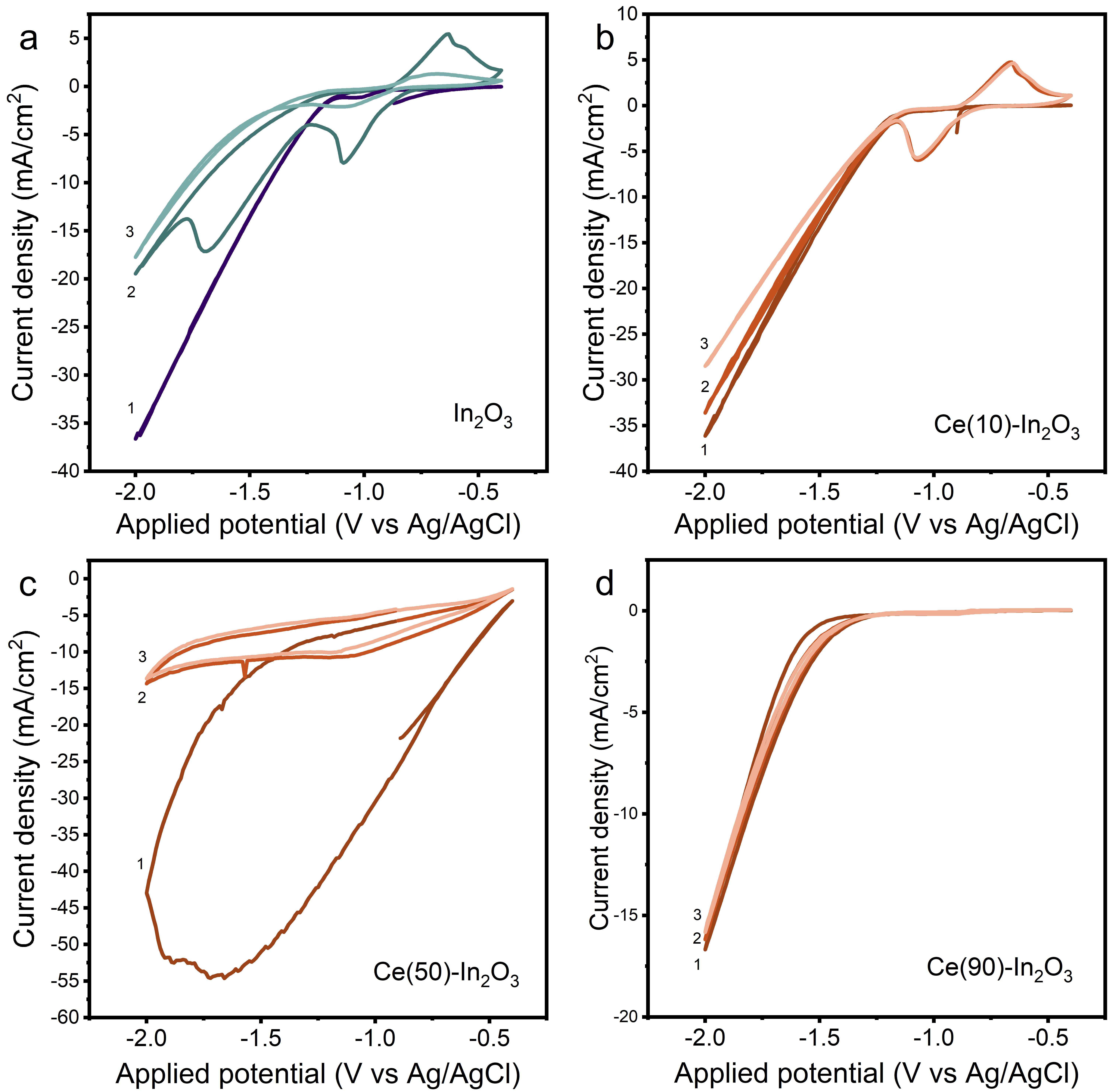


Figure S17: Cyclic voltammetry on pure In_2_O_3_ (a) and Ce-In_2_O_3_ (b-d) catalysts on a GDE during operando XAS analysis in CO_2_ saturated 0.5 M KHCO_3_. A constant CO_2_ flow of 5 ml/min was applied. The potential was swept between -0.4 V and -2.0 V vs Ag/AgCl at a scan rate of 10 mV/s. The In_2_O_3_ reduction and re-oxidation occur at an onset potential of -0.9 V, peaking at -1.1 V and -0.7 V respectively at this scan rate.

*
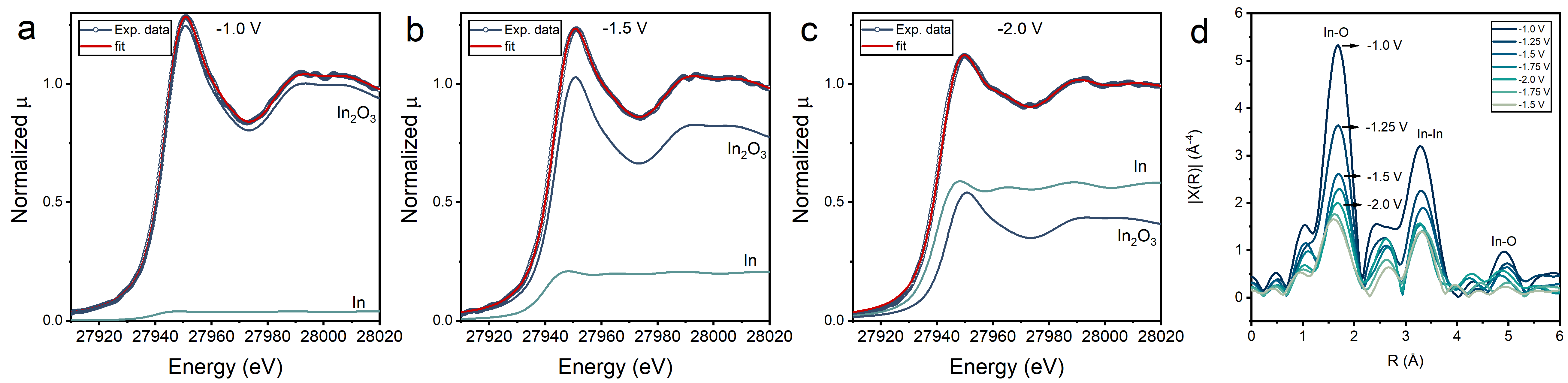
*

Figure S18: Linear combination fitting (LCF) on the XANES spectral region of In_2_O_3_ during the first reduction cycle at a potential of -1.0 V (a), 1.5 V (b), and -2.0 V (c) in the forward scan direction and -1.5 V in the subsequent reverse scan direction.

*
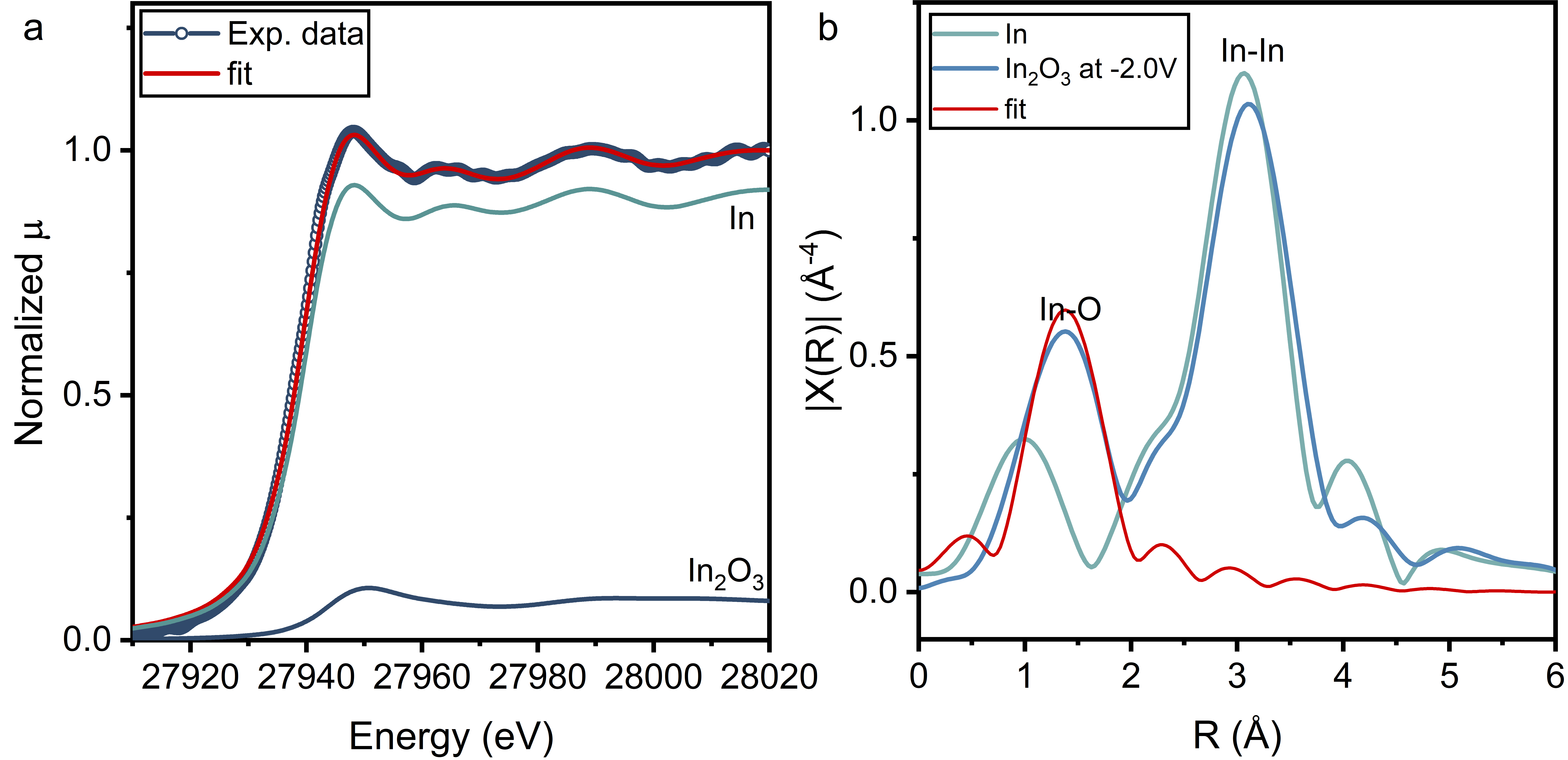
*

Figure S19: (a) LCF on the XANES spectral region of In_2_O_3_ during chrono amperometry at -2.0 V vs Ag/AgCl after 3 CVs. (b) The corresponding k^3^ weighted FT-EXAFS spectra (In K-edge) of In_2_O_3_ and reference metallic In. A small contribution of the In-O shell confirms the presence of a remaining InO_x_ phase during CO_2_ electroreduction.

*
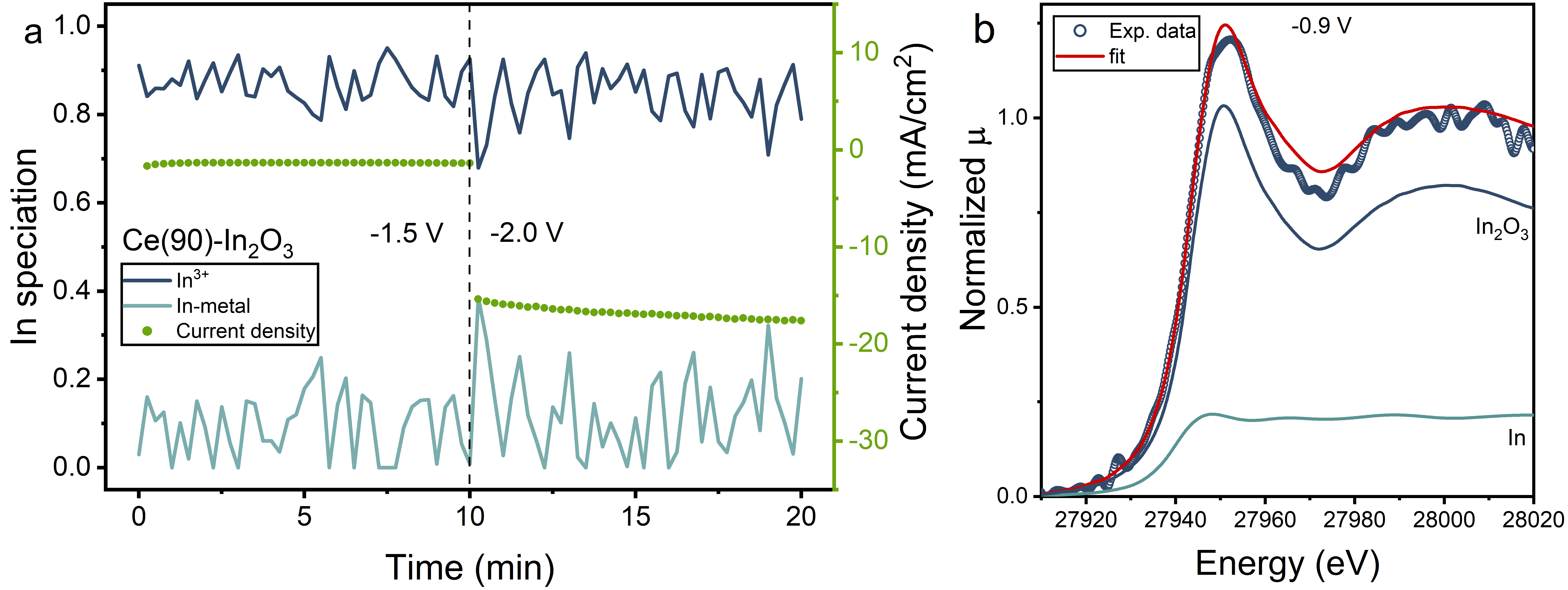
*

Figure S20: (a) Time resolved evolution of In speciation determined by LCF on the XANES spectral region of Ce(90)-In_2_O_3_ during chrono amperometry at -1.5 V and -2.0 V vs Ag/AgCl after 3 CVs. (b) LCF on the XANES spectral region of pure Ce(90)-In_2_O_3_ before cyclic voltammetry at a starting potential of -0.9 V.

*
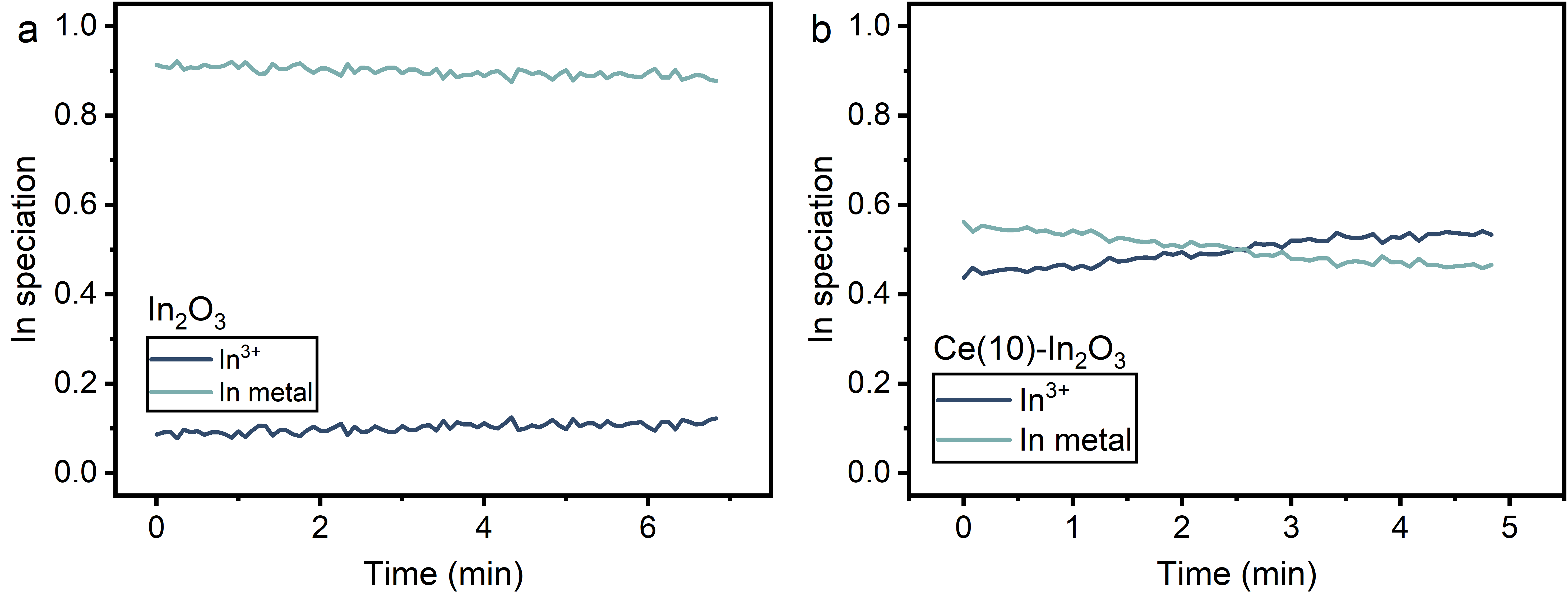
*

Figure S21: (a) Time resolved evolution of In speciation determined by LCF on the XANES spectral region of In_2_O_3_ during OCP after 3CVs and 20 min chrono amperometry. (b) Time resolved evolution of In speciation determined by LCF on the XANES spectral region of Ce(10)-In_2_O_3_ during OCP after 3CVs and 20 min chrono amperometry.

# References

(1) Wissink, T.; van de Poll, R. C. J.; Figueiredo, M. C.; Hensen, E. J. M. Stability of In2O3 Nanoparticles in PTFE-Containing Gas Diffusion Electrodes for CO2 Electroreduction to Formate. *J. CO2 Util.* **2023**, *67* (August 2022), 102331.

(2) Skála, T.; Šutara, F.; Prince, K. C.; Matolín, V. Cerium Oxide Stoichiometry Alteration via Sn Deposition: Influence of Temperature. *J. Electron Spectros. Relat. Phenomena* **2009**, *169* (1), 20–25.

(3) Kibis, L. S.; Titkov, A. I.; Stadnichenko, A. I.; Koscheev, S. V.; Boronin, A. I. X-Ray Photoelectron Spectroscopy Study of Pd Oxidation by RF Discharge in Oxygen. *Appl. Surf. Sci.* **2009**, *255* (22), 9248–9254.

(4) Muravev, V.; Parastaev, A.; van den Bosch, Y.; Ligt, B.; Claes, N.; Bals, S.; Kosinov, N.; Hensen, E. J. M. Size of Cerium Dioxide Support Nanocrystals Dictates Reactivity of Highly Dispersed Palladium Catalysts. *Science (80-. ).* **2023**, *380* (6650), 1174–1179.

(5) Toyoshima, R.; Yoshida, M.; Monya, Y.; Kousa, Y.; Suzuki, K.; Abe, H.; Mun, B. S.; Mase, K.; Amemiya, K.; Kondoh, H. In Situ Ambient Pressure XPS Study of CO Oxidation Reaction on Pd(111) Surfaces. *J. Phys. Chem. C* **2012**, *116* (35), 18691–18697.

(6) Biesinger, M. C.; Payne, B. P.; Grosvenor, A. P.; Lau, L. W. M.; Gerson, A. R.; Smart, R. S. C. Resolving Surface Chemical States in XPS Analysis of First Row Transition Metals, Oxides and Hydroxides: Cr, Mn, Fe, Co and Ni. *Appl. Surf. Sci.* **2011**, *257* (7), 2717–2730.

(7) Biesinger, M. C.; Payne, B. P.; Lau, L. W. M.; Gerson, A.; Smart, R. S. C. X-Ray Photoelectron Spectroscopic Chemical State Quantification of Mixed Nickel Metal, Oxide and Hydroxide Systems. *Surf. Interface Anal.* **2009**, *41* (4), 324–332.

(8) Salunkhe, P.; Muhammed Ali, A. V.; Kekuda, D. Investigation on Tailoring Physical Properties of Nickel Oxide Thin Films Grown by Dc Magnetron Sputtering. *Mater. Res. Express* **2020**, *7* (1).

(9) Barroso-Bogeat, A.; Blanco, G.; Pérez-Sagasti, J. J.; Escudero, C.; Pellegrin, E.; Herrera, F. C.; Pintado, J. M. Thermocatalytic CO2 Conversion over a Nickel-Loaded Ceria Nanostructured Catalyst: A Nap-Xps Study. *Materials.* **2021**, *14* (4), 1–19.
